# A Comparative Analysis of Zinc Oxide as Nanoparticles and Its Bulk Form through In Vitro Toxicogenetics and In Vivo Zebrafish Embryotoxicity Models

**DOI:** 10.1021/acsomega.6c07375

**Published:** 2026-09-22

**Authors:** Carlos Fernando Araujo-Lima, Magno Maciel-Magalhães, Marcela Correia da Rocha Honorato, Hana de Jesús Galiza Gomes, Pedro Paulo Saldanha Coimbra, Nayara Cecília do Couto Guedes, Thiago dos Santos Cabral-Rocha, Lays Souza, Bárbara Verena Dias Galvão, Beatriz Rodrigues Canabarro, Gabriele Fátima de Souza, Thais Morais de Brito, Otniel Freitas-Silva, Renata Jurema Medeiros, Beatriz Ferreira de Carvalho Patricio, Israel Felzenszwalb

**Affiliations:** † Universidade Federal do Estado do Rio de Janeiro (UNIRIO), Laboratório Integrado de Mutagênese Ambiental (LIMA), Rio de Janeiro 20211-010, RJ, Brazil; ‡ Universidade do Estado do Rio de Janeiro (UERJ), Laboratório de Mutagênese Ambiental (LABMUT), Rio de Janeiro 20551-030, RJ, Brazil; § Universidade Federal do Estado do Rio de Janeiro (UNIRIO), Laboratório de Inovação Farmacêutica e Tecnológica (LIFT), Rio de Janeiro 20211-010, RJ, Brazil; ∥ Universidade Federal do Estado do Rio de Janeiro (UNIRIO), Programa de Pós-Graduação em Alimentos e Nutrição (PPGAN), Rio de Janeiro 20211-010, RJ, Brazil; ⊥ 37903 Fundação Oswaldo Cruz (FIOCRUZ), Instituto Nacional de Controle de Qualidade em Saúde (INCQS), Plataforma de Ensaios Toxicológicos em Peixes (FIOFISHTOX), Rio de Janeiro 21040-900, Brazil; # Centro Brasileiro de Pesquisas Físicas (CBPF), Rio de Janeiro 22290-180, Brazil; ∇ Empresa Brasileira de Pesquisa Agropecuária (EMBRAPA), Rio de Janeiro 23020-470, RJ, Brazil

## Abstract

Zinc oxide nanoparticles (ZnO-NP) are widely used due to their enhanced biological activity compared to the bulk counterpart (ZnO-BK), raising concerns about potential toxic effects on living organisms and the environment. This study investigated the toxicity of ZnO-NP and ZnO-BK using alternative in vitro and in vivo models. The *Salmonella*/Microsome assay (±metabolic activation) was conducted according to the Organisation for Economic Co-operation and Development (OECD) guidelines. HepG2 cell viability and genotoxicity were assessed using the WST-1 and cytokinesis-block micronucleus (CBMN) assays. Acute toxicity was evaluated using the fish embryotoxicity (FET) test in zebrafish (*Danio rerio*) embryos. Both forms induced mutagenic responses in TA98 and TA102 strains, while ZnO-BK showed mutagenicity in TA100 only in the absence of metabolic activation. In HepG2 cells, both forms induced dose-dependent cytotoxicity in the WST-1 model, with ZnO-BK exhibiting greater cytotoxic potency. The CBMN assay revealed higher cytogenetic damage for ZnO-BK, including micronuclei, nuclear buds, and nucleoplasmic bridges, while both forms exhibited dose-dependent cytotoxic and cytostatic effects. In vivo, ZnO-NP exerted a more pronounced impact on embryonic development, altering eye diameter and body size compared to ZnO-BK. Overall, both forms demonstrated significant toxicological effects, highlighting their relevance for regulatory toxicogenetics and environmental safety.

## Graphical abstract

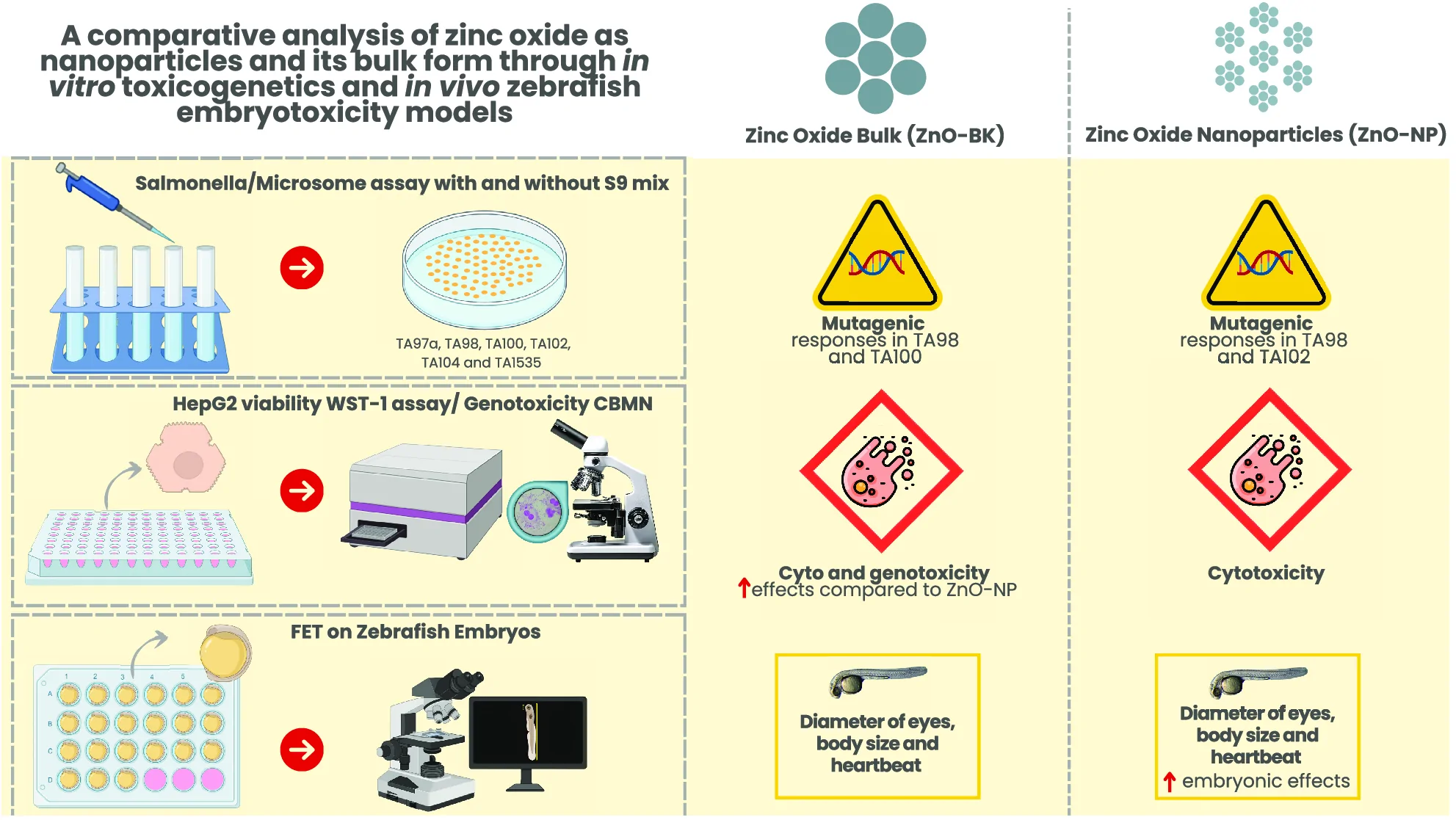

## 1 Introduction

Nanoparticles (NP) are defined as nanoscale objects (1–100 nm) possessing relatively isometric dimensions.^1^ They may vary in shape, structure, and composition and are typically classified as organic, carbon-based, or inorganic.^2,3^ Their synthesis may occur through top-down and bottom-up approaches. In the top-down approach, the bulk material is progressively reduced to nanometric particles through physical and mechanical processes, whereas in the bottom-up approach, structures are formed from atoms that assemble into clusters to generate NPs.^4^ Due to the high surface area-to-volume ratio and quantum-size effects, NPs exhibit physicochemical and biological properties distinct from their bulk counterparts, including greater reactivity, stability, and mechanical resistance, as well as specific optical and magnetic properties.^4,5^ These characteristics enable a wide range of applications that extend from research to industry. In biomedicine, they stand out in the development of diagnostic tools and new therapies, especially in drug delivery.^2^

Inorganic NPs tend to exhibit biocompatibility and hydrophilicity, as well as greater stability than organic NPs.^6^ They can be classified into metallic and metal oxide-based categories, the latter being formed from the combination of metal cations and oxygen anions, whose electrostatic interactions stabilize ionic bonds.^3^ Due to their ability to interact with cellular structures, metal oxide NPs are frequently associated with increased reactivity and efficiency.^7^ Among them, zinc oxide nanoparticles (ZnO-NP) have emerged as a promising candidate due to their low cost and properties such as chemical stability, conductivity, and the ability to block ultraviolet radiation.^8^ Classified as “Generally Recognized as Safe” (GRAS) by the United States Food and Drug Administration (FDA), ZnO is widely used in commercial products such as cosmetics and sunscreens, and exhibits several biological activities, including antibacterial, antitumoral, and anti-inflammatory effects, as well as potential applications in biosensors, biological labeling, and therapeutic carriers.^9,10^ Notably, ZnO has been explored across various industrial sectors, including electronics, textiles, agriculture, and environmental remediation, showcasing proven efficiency in water purification, pollution control, and air quality improvement.^11,12^

However, the scaling of ZnO-NP production, coupled with its release into the environment, has raised concerns about risks to health and the ecosystem.^8^ Global manufacturing and consumption are steadily expanding, with estimated discharges ranging from 550 to 5550 tons annually via domestic and industrial wastes. Prolonged exposure arising from everyday consumer goods may lead to chronic toxicity.^2,13^ The primary exposure routes encompass inhalation, dermal contact, and ingestion.^14^ Upon crossing the cutaneous barrier, these nanomaterials may trigger allergic responses, while in the lungs, they are linked to pulmonary diseases. In addition, these NPs may trigger neurotoxicity by crossing the blood-brain barrier, inducing cell damage and death via the generation of reactive oxygen species (ROS) and oxidative stress.^15–17^ In ecological contexts, ZnO-NPs disrupt soil microbial diversity, inhibit algal growth, and exert toxic effects on fish, driven by bioaccumulation, ultimately magnifying human health risks through the food web.^8,11^

The comparison between ZnO-NPs and their bulk counterparts (ZnO-BK) demonstrates biological alterations associated with shape, structural size, and kinetics, highlighting the influence of the nanoscale on physiological outcomes.^18,19^ Consequently, rigorous risk assessment is fundamental to ensuring their safe application.^12^ For this purpose, relying on standardized assays issued by regulatory bodies, such as the Organisation for Economic Co-operation and Development (OECD), is essential. Among these, the Salmonella/microsome bacterial reverse mutation assay (TG471),^20^ commonly known as the Ames Test, the in vitro mammalian cell micronucleus test (TG487),^21^ and the fish embryotoxicity (FET) test (TG236)^22^ are particularly relevant tools for evaluating mutagenic potential, genotoxic damage, and embryotoxicity. Complementing these approaches, in vitro hepatic models offer crucial insights into liver-specific outcomes, given that the liver is a primary site of nanomaterial bioaccumulation following systemic exposure.^23^

Regarding in vivo assessment, zebrafish stand as a well-established model organism in (eco)­toxicology, widely adopted for aquatic environmental hazard and risk assessment due to their short reproductive cycle, year-round breeding, small size, and ease of laboratory maintenance.^24–26^ Specifically, the FET test exploits the natural transparency of zebrafish embryos to directly monitor a broad spectrum of toxic end points, ranging from those considered to be lethal effects (egg coagulation, absence of somite formation, tail bud detachment, and heartbeat cessation) to sublethal morphological alterations, including edema formation, hatching delay, uninflated swim bladder, and malformations of the eyes, mouth, or tail.^22,27,28^

Therefore, the main objective of this study is to evaluate the toxicogenetic effects of ZnO in both its bulk and nanoparticulate forms by integrating complementary in vitro and in vivo approaches, encompassing mutagenic potential in bacterial assays, genotoxic and cytogenetic damage in hepatic cells, and embryotoxicity assessed through a standardized fish toxicity test.

## 2 Materials and Methods

### 2.1 Samples

The ZnO-NP and ZnO-BK were purchased from Sigma-Aldrich (USA) and Dinâmica Química Contemporânea (Brazil), respectively, and kindly provided by Otniel Freitas Silva (Embrapa Agroindústria de Alimentos, Rio de Janeiro, Brazil). The samples were maintained at −80 °C and protected from light exposure.

### 2.2 Particle Size Analysis

The mean particle size and Polydispersity Index (PDI) were measured by dynamic light scattering for ZnO-NP using dynamic light scattering (Malvern Zetasizer Ultra Red, Worcestershire, U.K.). A stock solution (400 μg/mL) was produced in ultrapure water (Samtec, Brazil). Samples were prepared by diluting the stock in different media. Nanoparticle size measurements were performed using the backscatter detection configuration (173°) and side angle (90°) at 25 °C. The hydrodynamic diameter was calculated using the cumulants analysis. For ZnO, a refractive index of 2.00 and an absorption coefficient of 0.10 were used. The physicochemical parameters of the dispersants were defined according to the composition of each medium: ultrapure water (refractive index, 1.330; viscosity, 0.887 cP; dielectric constant, 78.5), DMEM (refractive index, 1.337; viscosity, 0.930 cP; dielectric constant, 78.0), E3 medium (refractive index, 1.334; viscosity, 0.89 cP; dielectric constant, 78.5), phosphate buffer (refractive index, 1.334; viscosity, 0.911 cP; dielectric constant, 78.6), and DMEM supplemented with 10% fetal bovine serum (FBS) (refractive index, 1.336; viscosity, 1.00 cP; dielectric constant, 78.0). After dilution, the samples were sonicated for 4 min at an amplitude of 20%. For each experimental condition, five independent measurements were performed, and the reported values represent the arithmetic mean of the five measurements. The mean particle size of ZnO-BK was measured by laser scattering using a Mastersizer 3000 (Malvern Instruments, U.K.) with a wet dispersion unit, Hydro MV. Ultrapure water was used as a dispersant. In the unit, moderate agitation was maintained, and the sample was added directly to the equipment containing the dispersant until the obscuration range was reached. The volume-based diameters of d10, d50, and d90 (sizes of particles for which 10%, 50%, and 90%, respectively, of the sample is below the given size), as well as the polydispersity (span index), were determined.

### 2.3 ζ-Potential (ZP)

For ζ-potential measurements, electrophoretic mobility was converted to ζ-potential using the corresponding dispersant properties, including viscosity, refractive index, and dielectric constant. The measurements were performed in Malvern Zetasizer Ultra Red (Worcestershire, U.K.), and the samples were prepared under the same conditions as for the size measurement. For each experimental condition, five independent measurements were performed, and the reported values represent the arithmetic means of the five measurements.

### 2.4 Morphology and Chemical Composition Analysis

Particles were dispersed on top of a carbon tape supported by an aluminum stub and then coated with gold using a high-vacuum sputter coater Q300T D Plus (Quorum), using 25 mA for 60s. The particle morphology and chemical composition were analyzed by scanning electron microscopy (SEM) coupled with energy-dispersive X-ray spectroscopy (EDS). All measurements were carried out at 15 kV of acceleration voltage using a Lyra3 (Tescan) SEM and an X-Max^N^ (OXFORD Instruments) EDS. The particle sizes of the samples ZnO-BK and ZnO-NP were measured using FIJI software.^29^

### 2.5 Bacterial Reverse Mutation Assay

The bacterial reverse mutation assay was performed according to previously established protocols^30,31^ and in compliance with the OECD Guideline No. 471.^20^

ZnO-NP and ZnO-BK stock solutions were prepared in dimethyl sulfoxide (DMSO, Sigma-Aldrich) at 10 and 20 mg/mL, respectively. These were subsequently diluted in series to obtain the following final concentrations: 0.005, 0.05, 0.5, 5, and 50 μg/plate. The assay employed *Salmonella enterica* serovar Typhimurium standard strains (TA97, TA98, TA100, TA102, TA104, and TA1535), in the presence and absence of exogenous metabolic activation (S9 mix). All samples were tested in triplicate. For the assays without metabolic activation (−S9), 500 μL of 0.2 M sodium phosphate buffer (pH 7.4) was added. For assays with metabolic activation (+S9), 500 μL of S9 mix was combined with 100 μL of bacterial culture (2 × 10^9^ cells/mL) and 100 μL of the sample. The negative control consisted of 1% DMSO. Positive controls for assays without exogenous metabolic activation included 9-aminoacridine (9-AA, 50 μg/plate, Sigma-Aldrich) for TA97a; 4-nitroquinoline-N-oxide (4NQO, 0.5 μg/plate, Sigma-Aldrich) for TA98; sodium azide (SA, 5 μg/plate, Sigma-Aldrich) for TA100 and TA1535; mitomycin C (MMC, 0.5 μg/plate, Sigma-Aldrich) for TA102; and methylmethanesulfonate (MMS, 250 μg/plate, Sigma-Aldrich) for TA104. In the S9 mix, 2-aminoanthracene (2AA, 5 μg/plate, Sigma-Aldrich) was used for all strains. The test tubes were incubated in a shaker at 37 °C and 60 rpm for 20 min. Then, 2 mL of top agar (0.7% agar, 0.5% NaCl, 0.05 mmol/L histidine, and D-biotin) at 40 °C was added to each tube, and the contents were poured onto minimal glucose agar plates. After incubation at 37 °C for 72 h, revertant colonies (*His* ^*+*^) were counted. The mutagenicity index (MI) was calculated, and mutagenicity was determined following three criteria: MI ≥ 3 for TA1535 and MI ≥ 2 for all the other strains; the presence of a dose–response relationship; and statistical significance assessed by one-way ANOVA (*p* < 0.05).

### 2.6 In Vitro Cytotoxicity

HepG2 cells (human hepatocellular carcinoma) were kindly provided by Dr. Daniele Palma de Oliveira (School of Pharmaceutical Sciences of Ribeirão Preto, University of São Paulo, Brazil). Cytotoxicity was assessed using the WST-1 assay according to established protocols.^32,33^ Cells were initially cultured in Dulbecco’s Modified Eagle Medium (DMEM) supplemented with 10% fetal bovine serum (FBS) and 1% penicillin/streptomycin (10 mg/mL) and maintained in a humidified incubator at 37 °C with 5% CO_2_. Upon reaching 80–90%, cells were seeded into 96-well plates at a density of 2 × 10^4^ cells/well. Cells were then exposed to ZnO-NP and ZnO-BK at concentrations ranging from 0.005 to 50 μg/mL for 24, 48, and 72 h (final volume 100 μL per well). A 1% DMSO solution was used as the negative control, and Triton X-100 was used as the positive control. After each incubation period, the supernatant was removed, and the wells were washed with phosphate-buffered saline (PBS). Subsequently, 100 μL of WST-1 reagent solution (prepared by adding 10 μL of reagent to 100 μL of fresh DMEM) was added to each well. Plates were incubated for 3 h, and absorbance was measured at 440 nm using a microplate reader. Cell viability was normalized to the 1% DMSO control, defined as 100% viability, with Triton X-100 representing maximum cytotoxicity. Statistical analysis was performed using one-way ANOVA followed by Tukey’s post hoc test, with *p* ≤ 0.05 considered significant.

### 2.7 In Vitro Cytokinesis-Block Micronucleus Assay (CBMN)

The CBMN assay was performed following the methodology described by Carrão-Dantas et al.^34^ HepG2 cells, seeded at a density of 2 × 10^4^ cells per well, were exposed to ZnO-NP and ZnO-BK at concentrations ranging from 3 to 24 μg/mL (in the range of the maximum dose was based on the LC_50_ of ZnO-NP in the WST-1 assay). Benzo-α-Pyrene 100 μg/mL (Sigma-Aldrich) served as a positive control. After 24 h of incubation at 37 °C in a 5% CO_2_, humidified atmosphere, cytokinesis was blocked by adding 3 μg/mL cytochalasin (Sigma-Aldrich) diluted in fresh medium. Cells were stained with diluted Giemsa solution (1:10) for 2 h, then analyzed by optical microscopy. To evaluate the nuclear division index (NDI) and the frequency of micronuclei (MN), 1000 cells per well (3000 cells per concentration) were examined under a bright-field microscope. Cells with intact, uniform nuclei and clear cytoplasmic boundaries were considered to exhibit a normal phenotypic morphology. Additionally, other cell abnormalities such as nuclear buds (NBUDs), nucleoplasmic bridges (NPBs), and cell death events (apoptotic and necrotic cells) were also recorded for each slide. Binucleated cells were observed using an optical microscope (Olympus C × 31-RTSF) at 40× magnification.^21^ The nuclear division index (NDI) and estimated survival rate were calculated using standard formulas from the literature for cytogenotoxicity assessment. All experiments were carried out in a triplicate and repeated at least twice. Statistical analysis for all tested parameters in this assay was performed using one-way ANOVA followed by Bonferroni’s post hoc test, with significance set at *p* ≤ 0.05.

### 2.8 Fish Embryo Acute Toxicity (FET) Test

The FET test was performed according to the guidelines established by the OECD,^22^ with minor modifications. The maintenance and mating of adult Zebrafish were conducted as previously described in Maciel-Magalhães et al.^35^ ZnO-NP samples were suspended in E3 medium (5 mM NaCl, 0.17 mM KCl, 0.33 mM CaCl_2_, 0.33 mM MgSO_4_)^36^ to obtain a stock solution of 58.4 μg/mL, which was subsequently diluted into four additional concentrations: 1.875, 3.75, 7.5, and 15 μg/mL. ZnO-BK was dissolved in pure DMSO and subsequently diluted into E3 medium to achieve the same concentrations, maintaining 1% DMSO in each tested dilution. For the assay, performed in triplicate, 20 fertilized embryos were used for each concentration in each replicate and were individually distributed into 24-well plates containing 2 mL of each diluted suspension. The four remaining wells on the plate were used as internal negative controls, containing E3 medium. For the negative control, a 24-well plate was prepared with E3 medium, with a maximum permissible mortality rate of 10%. The plates were incubated at 26 ± 1 °C in a calibrated and verified laboratory incubator (MMM Group, Ecocell, Monroe, WA). The tests were initiated at 1 h post-fertilization (hpf), and embryonic development was observed every 24 h, until the final time point at 96 hpf. At the end of the incubation period, the embryos were placed on microscope slides containing a drop of 15% low-viscosity sodium carboxymethylcellulose (Sigma-Aldrich) and, with the aid of a needle, were positioned in the best anatomical orientation to be photographed and filmed under a Leica microscope, model S9i, using LAS X 3.7.4 software, for approximately 15 s. Toxicity evaluation was based on four OECD-established criteria: embryo coagulation, absence of somites, failure of tail detachment, and absence of heartbeat. Using a semiquantitative scoring system described by Maciel-Magalhães et al.,^35^ points ranging from 0 to 4 were assigned to each larva to assess morphological and survival defects. Accordingly, 0 represented no visible alteration; 1 indicated only one nonlethal morphological alteration; 2 pointed to two nonlethal alterations; 3 corresponded to more than two nonlethal morphological alterations; and finally, 4 represented the occurrence of OECD-defined alterations or death. Additionally, the frequency of occurrence of each malformation was also determined for each group tested. Subsequently, 10 larvae per replicate (totaling 30 larvae) for each concentration were randomly chosen to measure their eye diameter, body length (from head to tail), and heart rate. For statistical analysis, data were tested for normality using the D’Agostino & Pearson test, followed by two-way ANOVA and Dunnett’s multiple comparisons test for results considered normal, and the Kruskal–Wallis test followed by Dunn’s multiple comparison test for cases considered non-normal, both considering *p* ≤ 0.05, in GraphPad Prism 9 software for MacOS.

### 2.9 Ethical Aspects

According to Information Note No. 01/2024 from the Brazilian National Council for the Control of Animal Experimentation (CONCEA), ethical licensing is not required when experiments are conducted exclusively on embryos up to the 120 h post-fertilization (hpf) period. The maintenance and mating of adult zebrafish (*D. rerio*) are licensed by FIOCRUZ Animal Ethics Committee (CEUA) under license number LW-29/23.

## 3 Results

### 3.1 Particle Characterization

The mean sizes of ZnO-BK and ZnO-NP are shown in Tables 1 and 2, respectively. The ZnO-NPs exhibited a Z-average, polydispersity index (PDI), and a ζ-potential that varied according to the media in which the nanoparticles are suspended. The bulk samples are in the micrometer range, with at least 50% below 5 μm. However, the span value (21.099) indicates a heterodisperse material.

**Table 1:** Size of ZnO-BK Measured by Laser Scattering

	Particle Size			
Sample	D10 (μm)	D50 (μm)	D90 (μm)	Span
ZnO-BK	1.31	3.98	85.3	21.099

**Table 2:** Size of ZnO-NP Measured by Dynamic Light Scattering and ζ-Potential

	Z-average (nm)	PDI	Z-average (nm)	PDI		
Sample	174°	174°	90°	90°	Concentrationμg/mL	ZP (mV)
Ultrapure water	232.5	0.3658	266.7	0.5127	50	–41.75
Phosphate buffer	2570	1	664.9	0.8596	50	-
Phosphate buffer	616.5	0.4231	761.5	0.5767	100	–27.05
DMEM	470.5	0.3722	720.2	0.5529	50	–13.37
DMEM + SFB	379.1	0.4623	284.2	0.5545	50	–15.74
E3 Media	289	0.4305	533	0.7186	50	–15.41

The particles’ morphology and size were identified using SEM analysis, while the chemical composition was obtained using EDS analysis (Figure 1). ZnO-BK exhibited two distinct morphologies: an elongated one, like a nanorod with approximately 87 nm in diameter, and an equi-axed and faceted one, while the ZnO-NP presented equi-axed nanoparticles. Both samples presented high purity, demonstrated in the EDS mapping, with only Zn and O identified as belonging to the sample. Other elements encountered were related to the sample support and preparation for SEM analysis (Figure S1).

**Figure 1:**
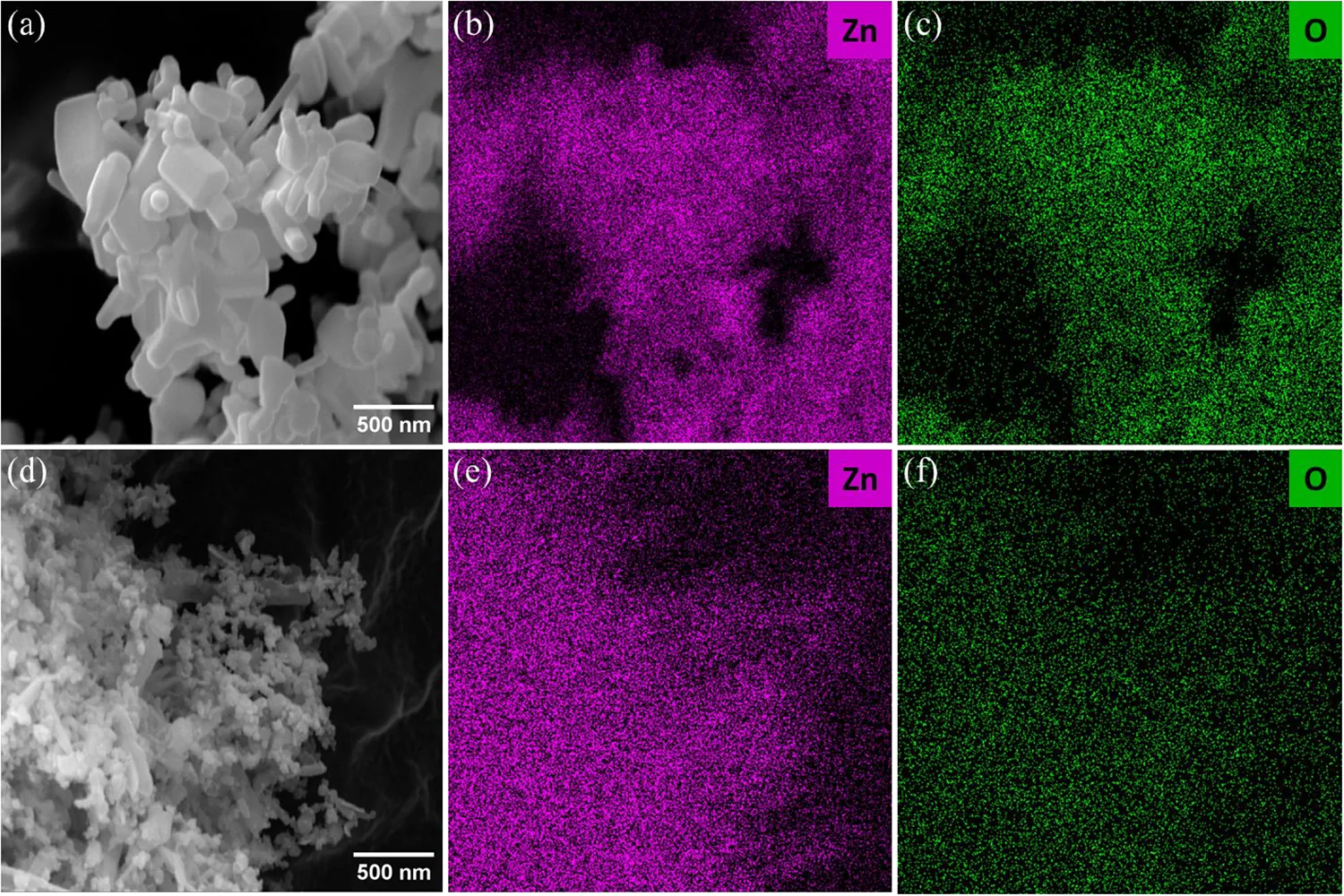
SEM images of zinc oxide bulk particles (ZnO-BK) (a), together with EDS Zn (b) and O (c) maps in comparison to its nanometric form (ZnO-NP) (d), as well as its EDS Zn (e) and O (f) maps.

The mean sizes of ZnO-BK and ZnO-NP found using SEM image analysis were smaller than those obtained with dynamic light scattering due to the observed particle agglomerations, which could be identified in an easier way using the images. Although the sizes were different for both samples, we also observed a broader dispersion in ZnO-BK particle size than ZnO-NP using this method (Figure 2). While the ZnO-BK exhibited a mean particle size of 275 nm with a standard deviation of 125 nm, ZnO-NP exhibited a mean particle size of 78 nm with a standard deviation of 48 nm (Table S1). Measurements and statistics are shown in the Supporting Information (Figure S2).

**Figure 2:**
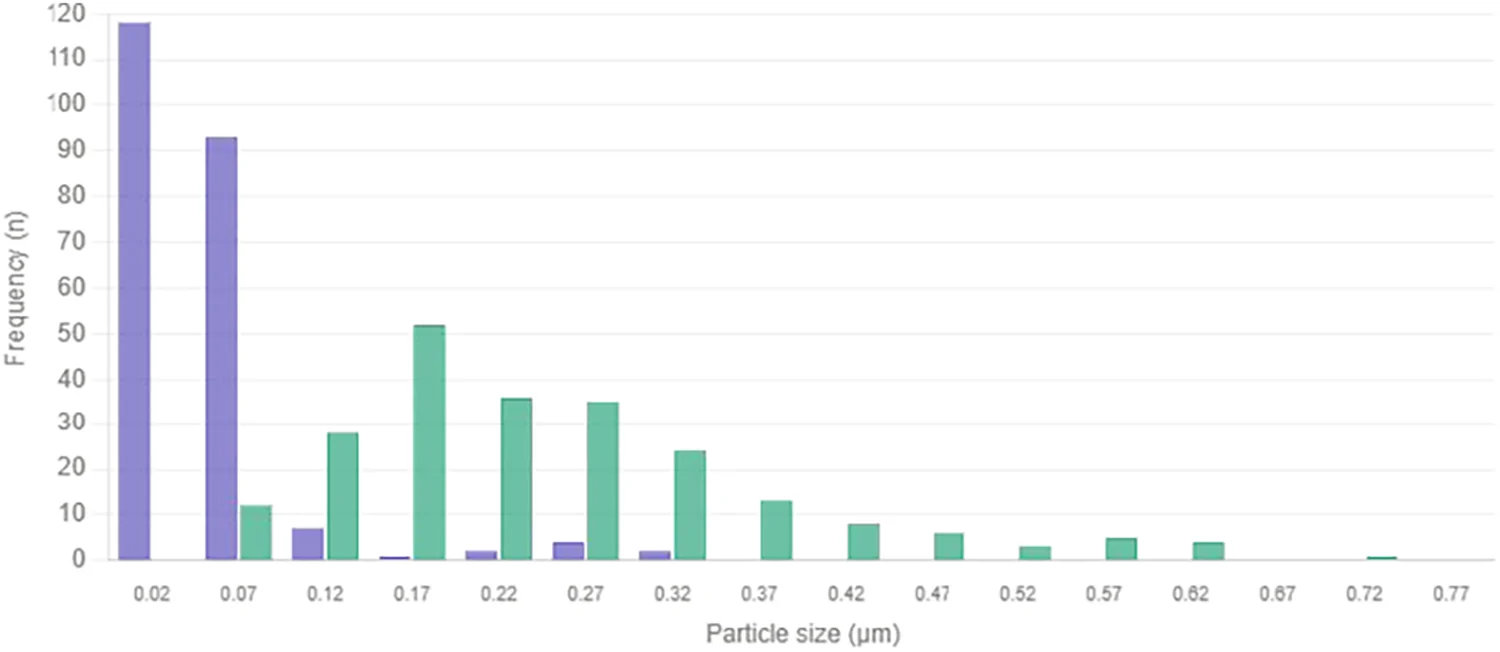
Particle size histogram measured using SEM images of ZnO-BK (green) and ZnO-NP (purple).

### 3.2 Bacterial Reverse Mutation Assay (Ames Test)

Both ZnO-NP and ZnO-BK induced mutagenicity in *S. Typhimurium* strains, regardless of metabolic activation (Table 3). In the absence of metabolic activation (-S9), ZnO-NP induced mutagenicity in TA98 at 50 μg/plate and in TA102 at 5 μg/plate and 50 μg/plate. ZnO-BK induced mutagenicity in TA98 above 0.5 μg/plate and in TA102 at 50 μg/plate. In the presence of metabolic activation (+S9), mutagenic effects were observed in TA98 for both samples at the lowest concentrations. For both samples, the reduction in MI observed in TA98 at the highest concentrations suggests that cytotoxic effects may have masked a stronger mutagenic response. Regarding TA102, ZnO-BK and ZnO-NP induced mutagenicity at all tested concentrations, with MI values ranging from 2.2 to 2.5, displaying a slight upward trend followed by stabilization at higher doses.

**Table 3:** Mutagenic Index and Number of Revertants of *S. enterica* Serovar Typhimurium at the Bacterial Reverse Mutation Assay^a^

		–S9				+S9			
		ZnO-NP		ZnO-BK		ZnO-NP		ZnO-BK	
Strain	μg/plate	M.I.	*His* ^*+*^ *±* SD	M.I.	*His* ^*+*^ *±* SD	M.I	*His* ^*+*^ *±* SD	M.I	*His* ^*+*^ *±* SD
**TA97a**	0	1.0	165 ± 5	1.0	165 ± 5	1.0	168 ± 4	1.0	168 ± 4
	0.005	0.3	47 ± 3	0.2	37 ± 4	0.3	52 ± 4	0.3	46 ± 4
	0.05	0.4	58 ± 3	0.4	60 ± 8	0.4	60 ± 6	0.4	58 ± 5
	0.5	0.4	68 ± 1	0.4	67 ± 3	0.4	68 ± 2	0.4	67 ± 2
	5	0.4	71 ± 4	0.5	78 ± 10	0.5	72 ± 2	0.4	71 ± 3
	50	0.5	83 ± 9	0.5	87 ± 8	0.6	91 ± 2	0.5	80 ± 4
**TA98**	0	1.0	30 ± 2	1.0	30 ± 2	1.0	35 ± 4	1.0	35 ± 4
	0.005	1.8	55 ± 6	1.2	36 ± 6	**2.1**	**75 ± 4**	**2.1**	**75 ± 3**
	0.05	1.9	56 ± 4	1.4	43 ± 9	1.9	67 ± 1	**2.0**	**69 ± 1**
	0.5	1.7	52 ± 5	**2.5**	**75 ± 9**	1.7	59 ± 2	1.9	67 ± 2
	5	1.8	54 ± 6	**3.0**	**90 ± 8**	1.6	55 ± 5	1.3	46 ± 5
	50	**2.4**	**72 ± 9**	**2.0**	**59 ± 8**	1.5	54 ± 4	1.0	36 ± 1
**TA100**	0	1.0	144 ± 8	1.0	144 ± 7.5	1.0	146 ± 11	1.0	146 ± 11
	0.005	0.2	31 ± 4	0.2	26 ± 3.6	0.1	20 ± 5	0.2	22 ± 4
	0.05	0.3	39 ± 8	0.2	30 ± 6.1	0.2	26 ± 7	0.2	27 ± 4
	0.5	0.2	32 ± 4	0.2	35 ± 5.9	0.2	25 ± 4	0.2	27 ± 2
	5	0.2	30 ± 3	0.3	38 ± 6.1	0.3	37 ± 3	0.3	39 ± 2
	50	0.2	30 ± 5	0.2	33 ± 5.8	0.3	37 ± 3	0.3	41 ± 2
**TA102**	0	1.0	324 ± 13	1.0	324 ± 13	1.0	461 ± 7	1.0	461 ± 7
	0.005	1.0	321 ± 4	0.7	222 ± 3	**2.2**	**1033 ± 6**	**2.2**	**1028 ± 6**
	0.05	1.0	323 ± 10	0.9	287 ± 9	**2.4**	**1127 ± 8**	**2.3**	**1078 ± 3**
	0.5	1.0	318 ± 10	1.7	554 ± 23	**2.4**	**1119 ± 7**	**2.4**	**1120 ± 7**
	5	**2.2**	**708 ± 38**	1.7	549 ± 45	**2.4**	**1122 ± 6**	**2.5**	**1169 ± 4**
	50	**2.7**	**879 ± 3**	**2.0**	**636 ± 14**	**2.5**	**1132 ± 7**	**2.4**	**1119 ± 13**
**TA104**	0	1.0	392 ± 10	1.0	392 ± 10	1.0	396 ± 5	1.0	396 ± 5
	0.005	0.4	155 ± 12	0.6	224 ± 8	0.4	166 ± 5	0.4	157 ± 3
	0.05	0.5	179 ± 7	0.6	232 ± 20	0.5	188 ± 2	0.5	186 ± 3
	0.5	0.8	318 ± 8	0.5	213 ± 11	0.5	193 ± 6	0.5	194 ± 6
	5	0.6	216 ± 10	0.6	248 ± 19	0.6	220 ± 5	0.6	225 ± 5
	50	0.5	194 ± 13	0.6	236 ± 11	0.6	225 ± 5	0.6	222 ± 8
**TA1535**	0	1.0	26 ± 4	1.0	26 ± 4	1.0	27 ± 2	1.0	27 ± 2
	0.005	0.3	8 ± 1	0.5	12 ± 2	0.5	13 ± 5	0.4	11 ± 2
	0.05	0.3	8 ± 2	0.6	16 ± 3	0.7	18 ± 2	0.6	15 ± 3
	0.5	0.5	12 ± 2	0.6	16 ± 2	0.7	18 ± 3	0.6	17 ± 3
	5	0.6	15 ± 2	0.7	17 ± 2	0.8	22 ± 3	0.8	21 ± 2
	50	0.6	15 ± 3	0.8	20 ± 2	1.1	29 ± 2	0.9	25 ± 5

a Subtitles: ZnO-NP: Zinc oxide nanoparticle; ZnO-BK: Bulk zinc oxide; −S9: absence of exogenous metabolism; +S9: presence of exogenous metabolism; M.I.: Mutagenicity index; SD: Standard deviation. In bold, positive results for mutagenicity, considering one-way ANOVA followed by Tukey’s post hoc test (*p* < 0.05). Positive controls without S9:9AA (50 μg/pl.) for TA97a, 585 ± 13 revertants; 4NQO (0,5 μg/pl.) for TA98, 600 ± 80 revertants; SA (5 μg/pl.) for TA100, 1200 ± 50 revertants; TA1535, 975 ± 48 revertants; MMC (0.5 μg/pl.) for TA102, 2130 ± 8 revertants; and MMS (250 μg/pl.) for TA104, 1120 ± 74 revertants. With S9:2AA (5 μg/pl.) for TA97a, 1000 ± 58; TA98, 1137 ± 9 revertants, TA100, 1400 ± 27 revertants and for TA1535 560 ± 23 revertants; for TA102, 1119 ± 13 revertants and for TA104 with 1486 ± 18 revertants.

### 3.3 Hepatic Cytotoxicity and Genotoxicity

The exposure of HepG2 cells to ZnO-NP and ZnO-BK resulted in concentration- and time-dependent cytotoxicity (Figure 3). As shown in Figure 3A, ZnO-NP induced a marked reduction in cell viability only at higher concentrations (>5 μg/mL), with no significant differences observed among 24, 48, and 72 h exposure times. In contrast, Figure 3B demonstrates that ZnO-BK exhibited higher cytotoxic potency, as evident from a leftward shift of the dose–response curves, with a more pronounced effect after 24 h of exposure.

**Figure 3:**
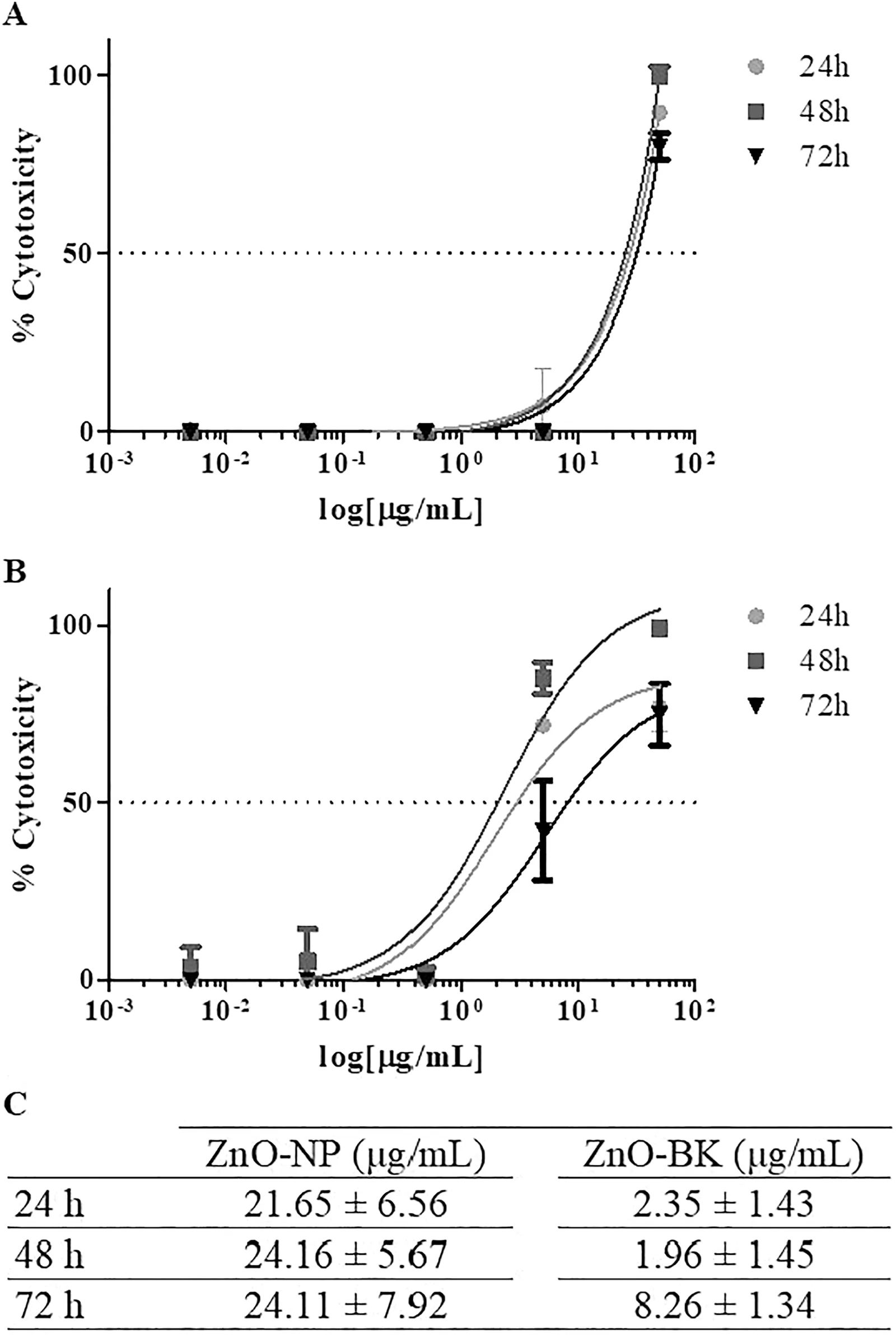
Hepatocytotoxicity of zinc oxide nanoparticles (ZnO-NP) in comparison to its bulk (ZnO-BK) form. HepG2 cells were incubated with ZnO-NP (A) and ZnO-BK (B) for 24, 48, and 72 h. After the incubations, cell viability by mitochondrial function (WST-1 assay) was measured. The lethal concentration of 50% of the cell population (LC_50_) values (C) were calculated by a nonlinear regression fit of dose–response (agonist behavior) curves with *R* ^2^ ≈ 0.96.

The calculated LC_50_ values (Figure 3C) confirmed this difference in cytotoxicity between the two forms. ZnO-NP presented relatively stable LC_50_ values across time points (21.65 ± 6.56 μg/mL at 24 h, 24.16 ± 5.67 μg/mL at 48 h, and 24.11 ± 7.92 μg/mL at 72 h). In contrast, ZnO-BK showed significantly lower LC_50_ values, particularly at 24 h (2.35 ± 1.43 μg/mL) and 48 h (1.96 ± 1.45 μg/mL), with a slight increase at 72 h (8.26 ± 1.34 μg/mL). The dose–response curves exhibited high goodness-of-fit (*R* ^2^ ≈ 0.96), reinforcing the robustness of the analysis. Overall, these results indicate that ZnO-BK is markedly more cytotoxic to HepG2 cells compared to ZnO-NP at equivalent concentrations.

Exposure to ZnO-NP induced limited genotoxic effects under the tested conditions (Figure 4A). The frequency of micronuclei (MN/1000 binucleated cells) remained comparable to the negative control across all concentrations, with no statistically significant increases. The Nuclear Division Index (NDI) showed a significant dose-dependent decrease (Figure 4B) following ZnO-NP exposure, indicating a mild-to-high cytostatic effect. Regarding other nuclear abnormalities, nucleoplasmic bridges (NPBs) (Figure 4D) were not significantly altered across the tested concentrations. In contrast, nuclear buds (NBUDs) (Figure 4C) exhibited a small but significant increase at 24 μg/mL (*p* < 0.05). Cell death analysis (Figure 4E) revealed a concentration-dependent increase in both apoptosis and necrosis, resulting in reduced survival rates.

**Figure 4:**
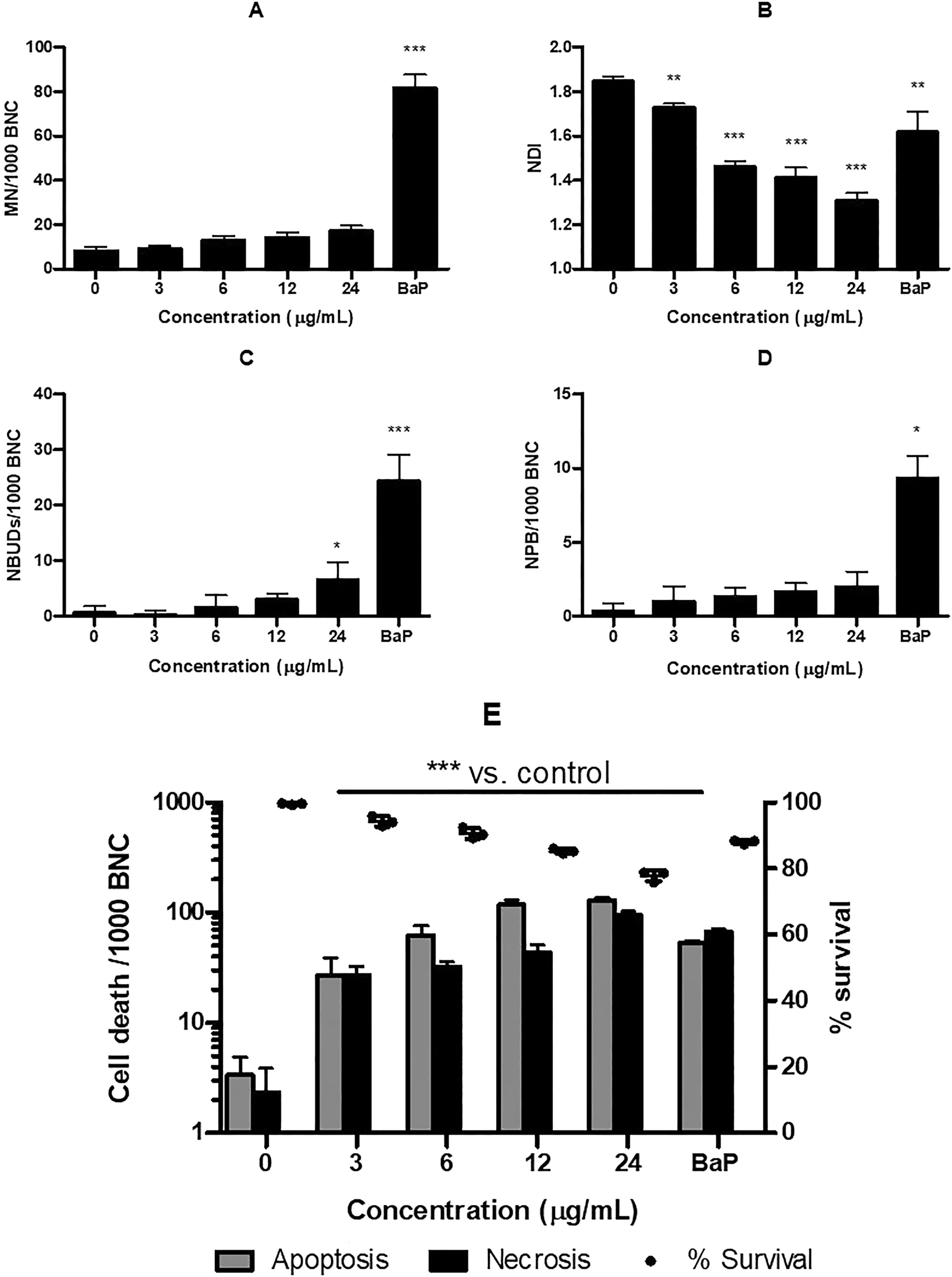
Hepatogenotoxicity of HepG2 cells exposed to ZnO-NP. (A) Micronuclei formation (MN), (B) nuclear division index (NDI), (C) nuclear buds (NBUDs), (D) nucleoplasmic bridges (NPBs), and (E) cell death (Apoptosis/Necrosis) and survival rate. The cells were exposed to ZnO-NP at concentrations of 3, 6, 12, and 24 μg/mL for 24 h, indicating potential genotoxic, cytotoxic, or cytostatic effects. BNC: Binucleated cells. BaP: Benzo-α-Pyrene 50 μg/mL. Three thousand cells were scored per treatment for each experiment (^∗^ *p* < 0.05, ^∗∗^ *p* < 0.01, ^∗∗∗^ *p* < 0.001 versus negative control; in triplicate, repeated at least twice, one-way ANOVA followed by a Bonferroni’s post hoc test).

In contrast to the nanoparticles, exposure to ZnO-BK resulted in pronounced genotoxic and cytotoxic effects (Figure 5). A concentration-dependent increase in micronucleus frequency was observed (Figure 5A), reaching statistical significance at 24 μg/mL. A clear reduction in NDI values was detected across increasing concentrations (Figure 5B), with significant decreases starting at 3 μg/mL and becoming more pronounced at higher doses. This finding indicates a marked inhibition of cell proliferation. Consistently, both NBUDs (Figure 5C) and NPBs (Figure 5D) were significantly elevated following ZnO-BK exposure. NBUD frequency increased significantly at concentrations above 12 μg/mL, with a strong effect at 24 μg/mL. Similarly, NPB formation was significantly higher at 12 and 24 μg/mL. Cell death analysis (Figure 5E) demonstrated a substantial increase in both apoptosis and necrosis in a concentration-dependent manner. Apoptosis predominated at intermediate concentrations, while necrosis became more evident at higher doses. These effects reflected a significant reduction in cell survival across all tested concentrations.

**Figure 5:**
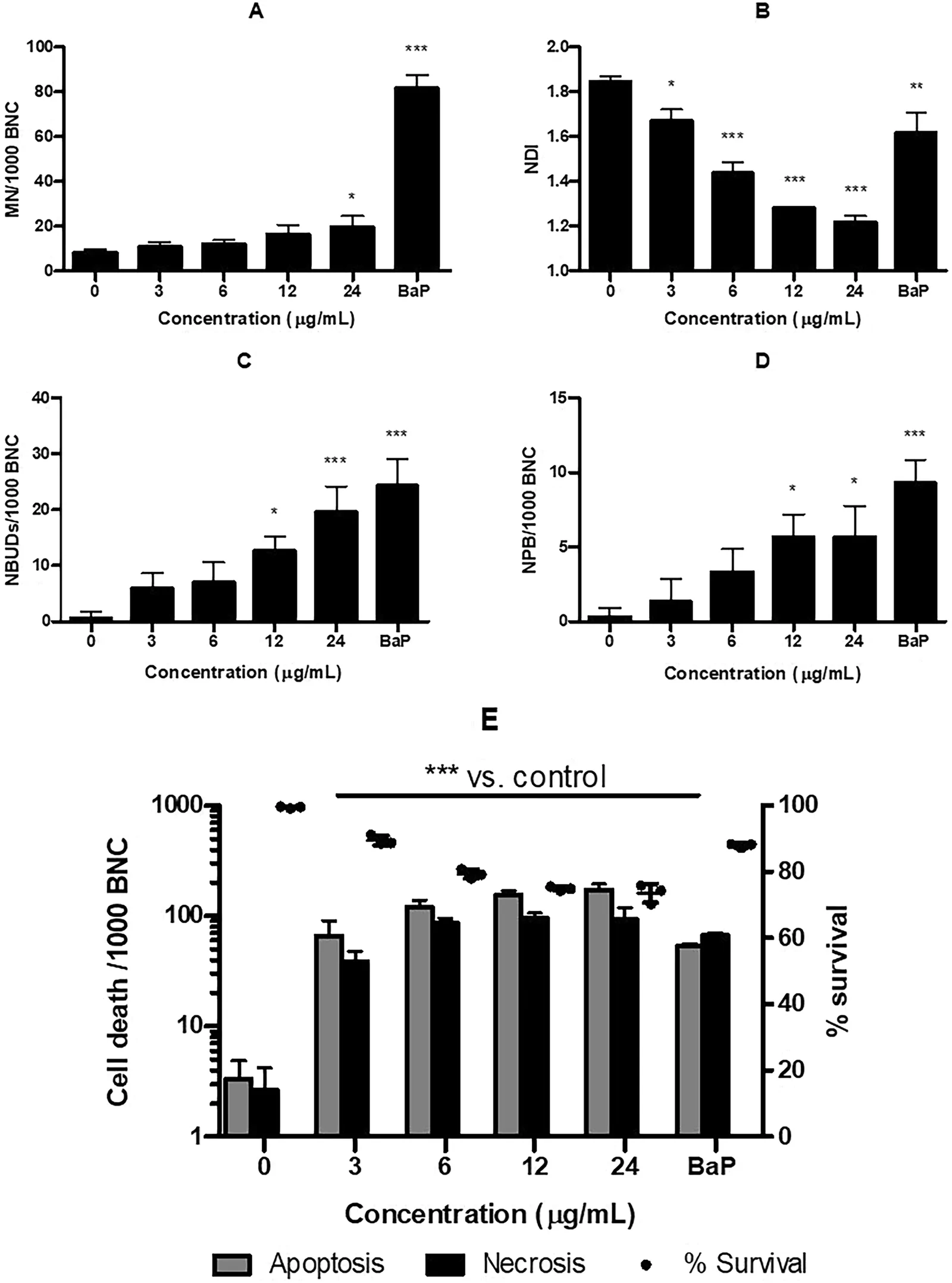
Hepatogenotoxicity of HepG2 cells exposed to ZnO-BK. (A) Micronuclei formation (MN), (B) nuclear division index (NDI), (C) nuclear buds (NBUDs), (D) nucleoplasmic bridges (NPBs), and (E) cell death (apoptosis/necrosis) and survival rate. The cells were exposed to ZnO-BK at concentrations of 3, 6, 12, and 24 μg/mL for 24 h, indicating potential genotoxic, cytotoxic, or cytostatic effects. BNC: Binucleated cells. BaP: Benzo-α-Pyrene 50 μg/mL. Three thousand cells were scored per treatment for each experiment (^∗^ *p* < 0.05, ^∗∗^ *p* < 0.01, ^∗∗∗^ *p* < 0.001 versus negative control; *n* = 3 in triplicate; repeated at least twice, one-way ANOVA followed by a Bonferroni’s post hoc test).

### 3.4 FET Test

Examples of images of zebrafish larvae treated with ZnO-NP or ZnO-BK, obtained at 96 hpf, are shown in Figure 6.

**Figure 6:**
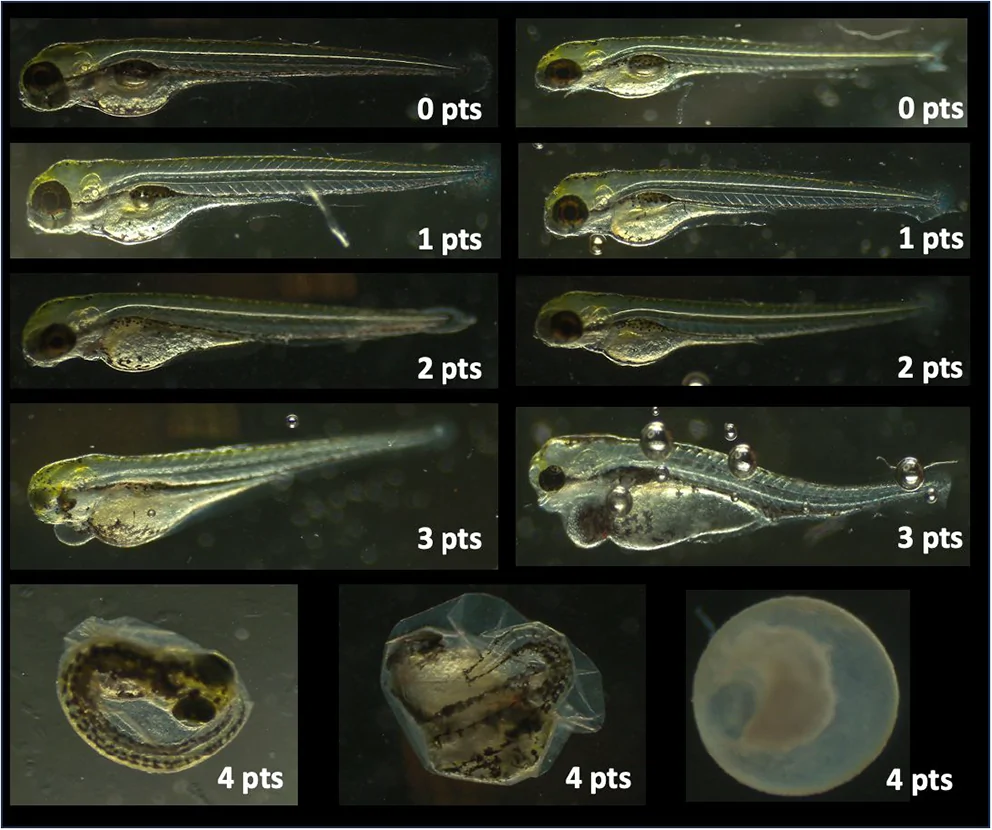
Examples of zebrafish larvae, photographed at 96 hpf, following exposure to ZnO-NP or ZnO-BK, and the scores assigned to each one. ZnO, zinc oxide; NP, nanoparticles; BK, bulk form; hpf, hours post-fertilization; pts, points.

Using the assigned points, a heat map was constructed (Figure 7) to visually illustrate the evolution of toxicity with an increasing concentration. The highest doses of both ZnO-NPs and ZnO-BK scored 4, differing from the negative control and the DMSO 1% control in the case of the BK form.

**Figure 7:**
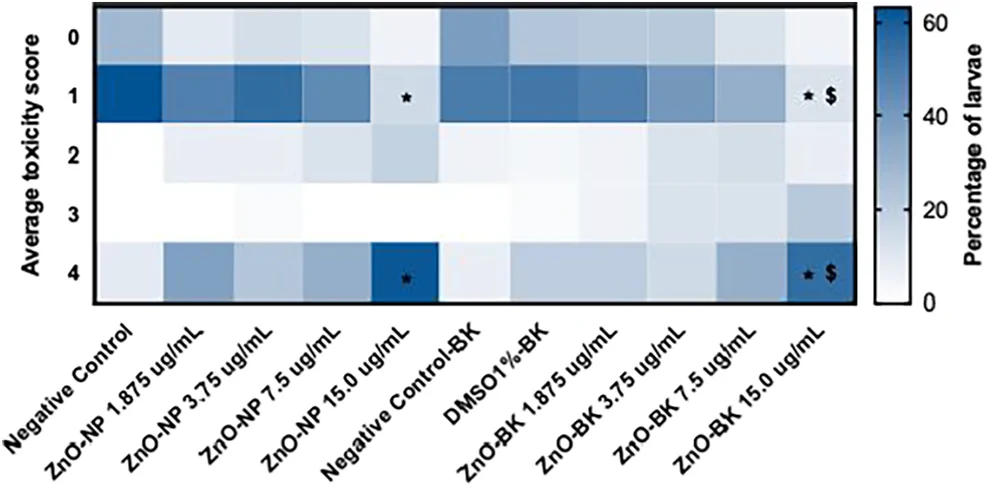
Percentages for each toxicity score obtained in the zebrafish embryotoxicity test at 96 hpf after exposure to ZnO-NP or ZnO-BK. ZnO-NP, zinc oxide nanoparticles; ZnO-BK, zinc oxide bulk form; hpf, hours post-fertilization. *n* = 20 per replicate (3), *p* ≤ 0.05 in Kruskal–Wallis test followed by Dunn’s multiple comparisons test; * represents differences between the sample and negative control (E3 medium); $ represents difference between the ZnO-BK sample and the DMSO 1% control.

In Figure 8, it is possible to observe the body size, eye diameter, and heartbeats per minute of the larvae at 96 hpf treated with different concentrations of ZnO-NP or ZnO-BK. A significant reduction in growth parameters was observed at the highest ZnO dose for both tested forms (Figure 8A,B).

**Figure 8:**
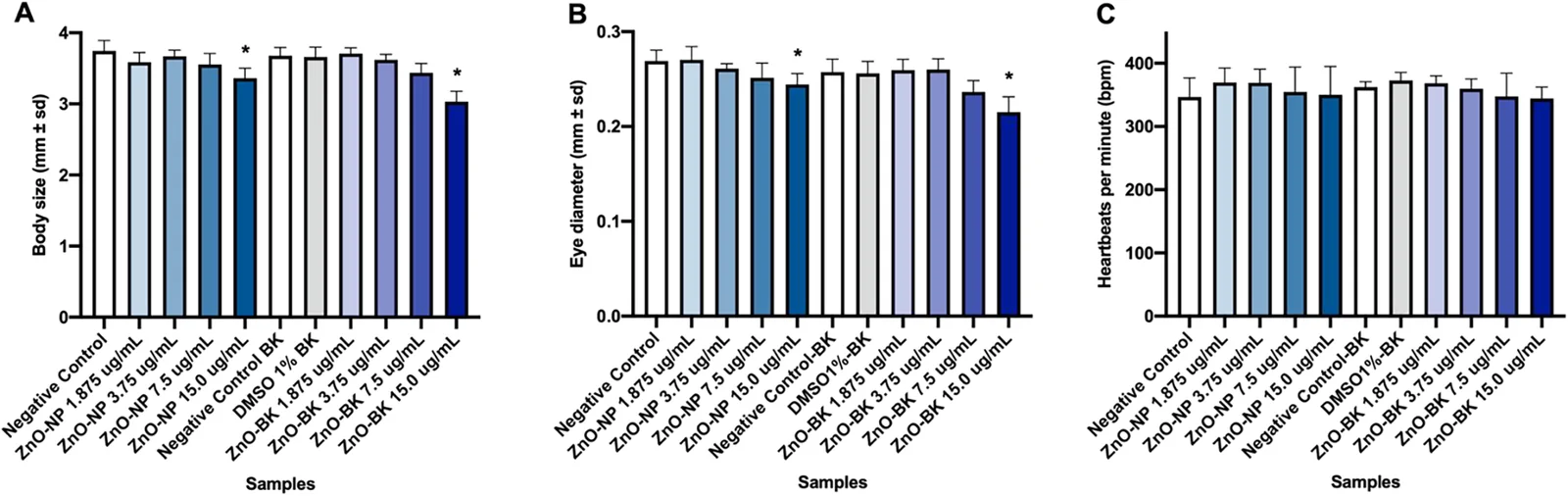
Body size (A), eye diameter (B), and heartbeats per minute (C) analysis of zebrafish larvae at 96 h post-fertilization incubated with ZnO nanoparticles (NP) or bulk (BK). Data shown as mean ± standard deviation. *n* = 10 in each group per replicate (3), *p* ≤ 0.05 in two-way ANOVA test followed by Dunnett’s multiple comparisons test; * represents the difference between the sample and the negative control (E3 medium).

Although isolated cases of pericardial edema were observed, statistical analysis revealed no significant difference in heart rate between the treated and control groups (Figure 8C). These findings suggest that neither ZnO-NP nor ZnO-BK exposure elicits substantial cardiotoxic effects in zebrafish larvae under the tested conditions.

Regarding the malformations observed in the FET assay, the frequency of occurrence of each one is described in Figure 9.

**Figure 9:**
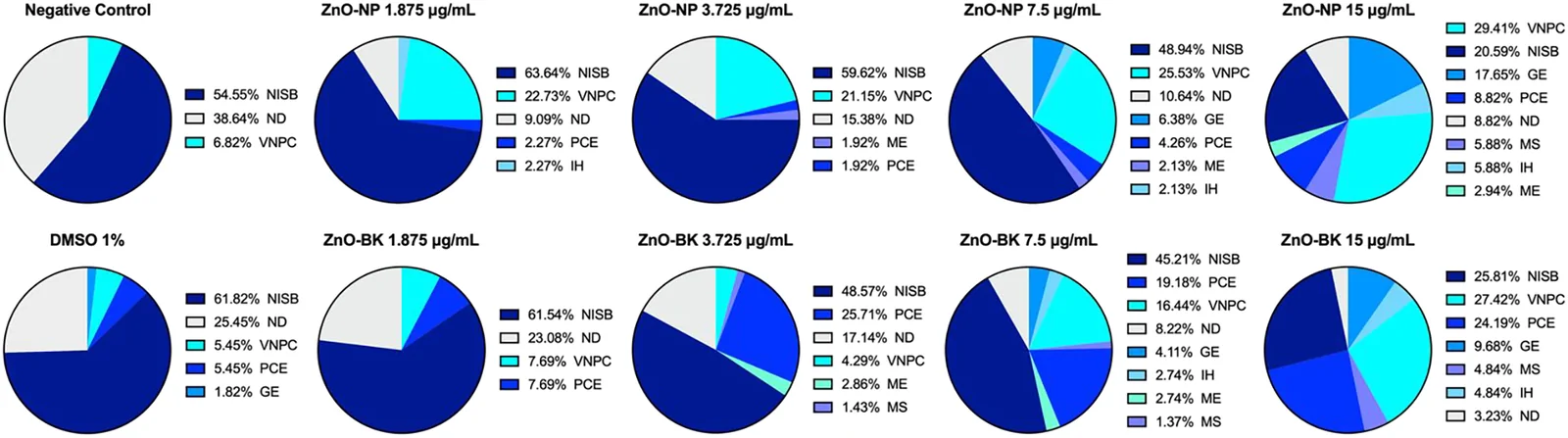
Frequency of occurrence of malformations in zebrafish larvae at 96 h post-fertilization incubated with ZnO nanoparticles (NP) or bulk (BK). NISB, noninflated swim bladder; VNPC, veal not/poorly consumed; PCE, pericardial edema; GE, general edema; IH, internal hemorrhage; MS, malformed spine; ME, malformed eye; ND, normally developed.

## 4 Discussion

The physicochemical characterization demonstrated that ZnO-NPs exhibited pronounced medium-dependent behavior, with substantial variations in hydrodynamic diameter, polydispersity index (PDI), and zeta potential (Table 2) across the different dispersants evaluated. These findings reinforce that the physicochemical properties of nanomaterials are dynamic rather than fixed characteristics and may change considerably, according to the composition of the surrounding environment.

In ultrapure water, ZnO-NPs presented comparatively smaller hydrodynamic diameters and a strongly negative ζ-potential, suggesting a relatively stable colloidal state under these conditions. The surface charge of ZnO is known to be highly influenced by the pH and ionic composition due to protonation and deprotonation reactions involving surface hydroxyl groups. Since the point of zero charge of ZnO is generally reported in the alkaline range, changes in dispersion conditions may substantially alter particle surface charge, colloidal stability, and dissolution behavior.^37,38^

The most pronounced physicochemical alterations were observed in phosphate buffer, where ZnO-NPs displayed marked increases in hydrodynamic diameter and PDI, indicating extensive aggregation. Such behavior is consistent with previous reports demonstrating strong interactions between phosphate ions and ZnO surfaces, resulting in surface modification, alteration of the electrical double layer, and reduced colloidal stability.^39,40^ In contrast, DMEM and E3 Media promoted less extensive aggregation despite also reducing electrostatic stabilization, suggesting that the medium composition plays a decisive role in determining nanoparticle behavior under biologically relevant conditions.

Interestingly, the addition of FBS resulted in a reduction in the measured hydrodynamic diameter, while the zeta potential remained relatively similar. This suggests that serum proteins can adsorb onto ZnO surfaces and form a protein corona, providing steric stabilization and modifying nanoparticle–nanoparticle interactions, which is particularly relevant for nanotoxicological studies because it may influence nanoparticle uptake, biodistribution, and biological responses.^41,42^

A similar pattern to that of DMEM was observed in E3 medium for ZnO-NPs. The relatively low absolute ζ-potential value indicates that electrostatic repulsion alone is unlikely to provide strong colloidal stabilization in this medium. Nevertheless, the hydrodynamic diameter remained lower than that in phosphate buffer, suggesting that the ionic composition of E3 influences ZnO aggregation differently. Overall, these findings demonstrate that ZnO nanoparticles undergo substantial medium-dependent changes in their hydrodynamic behavior and surface charge. Importantly, the physicochemical state of the nanoparticles under biological exposure conditions differs markedly from that observed in ultrapure water, emphasizing the importance of characterizing ZnO nanoparticles directly in the media used for toxicological assays.

An important feature of the present results was the difference in hydrodynamic diameter at 174° and 90° for the same dispersion medium, especially in phosphate buffer, DMEM, and E3 medium. Larger particles contribute disproportionately to the scattering signal and may therefore be more prominently detected at 90° than at the backscattering angle (174°). Consequently, differences between single-angle DLS measurements should not necessarily be interpreted as changes in the intrinsic nanoparticle size, but rather as evidence of heterogeneous particle populations and/or larger aggregates within the dispersion. The high PDI values observed in the present study support this interpretation.

Our findings demonstrate that both ZnO-NPs and their bulk counterparts induced mutagenic responses in *S. Typhimurium* strains TA102, TA98, and TA100, both with and without exogenous metabolic activation. The sensitivity of TA102 was particularly significant, as it carries an A-T base pair at the primary reversion site and is highly proficient at detecting oxidative mutagens.^20^ Moreover, its proficiency in the nucleotide excision repair system and the presence of the pKM101 plasmid enable it to detect cross-linking agents and severe DNA damage.^43^ Regarding strain TA98, which carries G-C base pairs at the primary reversion site,^44^ the results also exhibited susceptibility, suggesting that these materials trigger diverse mutagenic pathways. It is also worth noting that mutations observed in TA102, typically associated with base-pair substitutions, were also reported for copper oxide nanoparticles.^45,46^ Thus, our results reinforce that ZnO, regardless of its morphology (nano- or bulk), promotes a range of mutagenic events.

Although the release of metal ions due to particle dissociation upon cellular exposure has been described as the main mechanism of toxicity associated with metal oxide NPs,^47^ previous studies have indicated that Zn^2+^ ions released from nanoparticles alone may not exert a significant effect on cell viability.^48^ Instead, direct cellular contact with the particles may represent a key determinant of the cytotoxicity.

Our findings demonstrate that ZnO-NPs induced markedly lower genotoxic and cytostatic effects than ZnO-BK, as evidenced by higher frequencies of MN, NPBs, and NBUDs, alongside reduced NDI and cell survival. These results align with recent research highlighting that those physicochemical properties, such as high surface area and reactivity, drive the enhanced biological activity of ZnO-NPs compared to their bulk counterparts.^49^ The concomitant increase in micronuclei, nucleoplasmic bridges, and nuclear buds is indicative of chromosomal instability and DNA damage, corroborating previous reports describing ZnO-induced genomic alterations in biological systems.^50^ The mechanisms responsible for these effects were not directly assessed in this study. Nevertheless, ROS generation and Zn^2+^ release are widely recognized contributors to ZnO-mediated toxicity.^51,52^ Although nanoparticles are frequently assumed to be more toxic because of their greater surface area, recent evidence indicates that toxicity is not determined exclusively by nominal particle size and may depend on aggregation state, dissolution kinetics, effective cellular dose, and exposure conditions.^53–55^ Indeed, zinc ions themselves have been shown to induce concentration-dependent cytotoxic and genotoxic effects independently of their nanoparticulate form.^56^ Therefore, the greater cytogenetic damage observed after ZnO-BK exposure may reflect differences in particle behavior in the culture medium and in the effective dose reaching the cells rather than an intrinsic size-dependent effect alone. These findings reinforce the concept that physicochemical behavior under biological conditions is a stronger determinant of toxicity than nominal particle size itself.^17^

The zebrafish embryotoxicity tests revealed a contrasting pattern. At the highest tested concentration, both ZnO forms induced developmental toxicity, characterized by delayed hatching, increased malformation scores, reduced body length, and decreased eye diameter. However, the nanoparticulate form produced a more pronounced embryotoxic response, whereas no significant alteration of heart rate was observed. These findings agree with previous studies showing that ZnO exposure impairs embryonic development, induces growth retardation, and promotes concentration-dependent developmental toxicity in zebrafish embryos.^62–68^

During exposure, both materials formed visible aggregates in the E3 medium, with ZnO-BK exhibiting greater sedimentation on the bottom of the wells and around the chorion. The observed developmental effects may therefore be associated with a combination of particle aggregation, local Zn2+ release, and nanoparticle-chorion interactions. Delayed hatching and developmental abnormalities are consistent with mechanisms previously proposed for ZnO toxicity, including chorion surface blockage, reduced oxygen diffusion, and nanoparticle penetration through chorionic pores.^57–61^

In addition, several studies indicate that oxidative stress and Zn^2+^-mediated toxicity are central processes underlying ZnO embryotoxicity.^62–68^ Viewed in this context, the malformations, reduced body size, and decreased eye diameter observed in our FET at 15 μg/mL likely represent downstream manifestations of earlier oxidative and genomic disturbances described in these zebrafish studies. Mechanistically, our data on delayed hatching and increased malformation scores are compatible with both particle–chorion interactions (aggregation on the chorion surface, reduced oxygen diffusion, and potential nanoparticle penetration) and Zn^2+^-mediated toxicity, in line with reports that ZnO-NPs undergo substantial dissolution in embryo media and that dissolved Zn^2+^ can reproduce many of the observed effects.^65–67^

Thus, our results complement the existing literature by showing that both ZnO-NPs and ZnO-BK can induce significant developmental toxicity in zebrafish embryos under short-term exposure, while previous ELS, gene-DarT, and neurotoxicity studies demonstrate that ZnO can already compromise key molecular pathways and sensory functions at much lower concentrations. This integrated view underscores the importance of assessing ZnO nanotoxicity in zebrafish using a combination of structural, functional, and molecular endpoints to capture the full range of potential adverse outcomes.

The use of different vehicles was necessary because of the distinct dispersibility characteristics of the materials. ZnO-NPs remained suspended in E3 medium, whereas ZnO-BK required the use of 1% DMSO. The inclusion of a solvent control demonstrated that this concentration of DMSO did not significantly affect embryonic development, supporting the interpretation that the observed effects were attributable to ZnO exposure rather than to the vehicle itself.^69–72^

The distinct embryotoxic profiles of ZnO-NP and ZnO-BK may be explained by the complex interplay between particle aggregation and ion release kinetics. The observed sedimentation of aggregates on the chorion surface is a critical observation; as reported by Zhu et al.,^73^ this process can potentially create a localized microenvironment with high zinc concentrations and physical blockage of chorionic pores. While the chorion acts as a size-selective barrier, the literature suggests that nanoparticles within the 10–50 nm range may translocate into the perivitelline space.^74,75^ Moreover, the superior toxicity of the nanoparticulate form is hypothesized to be driven by its higher surface-to-volume ratio, which likely facilitates faster dissolution and ROS generation. This “dual action” mechanism combining physical particle interaction and Zn^2+^ ion toxicity is consistent with the findings of Brun et al.^76^ and Kteeba et al.,^77^ although direct measurements of ROS and dissolution kinetics were not the primary focus of this study.

An important outcome of this work is the apparent divergence between cellular and embryonic models. Although ZnO-BK induced stronger cytotoxic and cytogenotoxic effects in HepG2 cells, ZnO-NPs produced more pronounced developmental toxicity in zebrafish embryos. Rather than representing a contradiction, these findings indicate that ZnO toxicity is both endpoint- and system-dependent. In cultured cells, the aggregation state, sedimentation behavior, and effective cellular dose may favor the bulk material, whereas during embryonic development, the smaller particle size of ZnO-NPs may facilitate interactions with the chorion and enhance Zn^2+^ availability within the embryo microenvironment. Thus, biological responses cannot be accurately predicted solely from the nominal particle size.

Overall, the present results demonstrate that both ZnO-NPs and ZnO-BKs possess mutagenic and toxicological potential, although their biological effects differ according to the experimental model employed. The combined evidence from bacterial, cellular, and embryonic systems suggests that ZnO toxicity cannot be predicted exclusively by particle size but instead emerges from the interaction between material physicochemical properties, dispersion state, ion release, and the biological environment. Future studies directly evaluating ROS generation, Zn^2+^ dissolution kinetics, and nanoparticle uptake will be important to clarifying the mechanisms responsible for the distinct responses observed across biological systems.

## 5 Conclusion

The use of engineered nanoparticles is already a growing reality in a wide range of areas and is poised to play a transformative role. However, there is still no consensus on the short- and long-term impacts of this technology on humanity, the environment, and ecological balance.

Nanoscale toxicity and the negative impacts of nanomaterials on exposed individuals, industry professionals, and patients make nanomaterials a sensitive issue for the future of medicine. Research on the adverse effects of nanomaterials on human health has advanced with technological progress. To address these risks, it is essential to establish regulations and laws that ensure the safe use of these materials. Nanotoxicology is a field of study that, through scientific research using in vivo and in vitro methodologies, helps to minimize risks and provides fundamental information to support these regulations.

In this study, both types of methodologies were used to improve the effectiveness of the results. These analyses raised significant concerns about the safety of using ZnO in its nanomacrostructural forms. Our results highlight significant safety concerns: the Ames test confirmed mutagenic potential; the WST-1 assay demonstrated cytotoxicity, especially of ZnO-BK, culminating in metabolic impairment and cell death; and in vivo assays with zebrafish embryos revealed deleterious effects on morphological and neuronal development.

While these findings serve as critical indicators of potential risk rather than definitive evidence of human carcinogenicity, they underscore the need for caution. A deeper understanding of the physicochemical mechanisms of nanoparticle toxicity and their bulk counterparts is imperative for developing strategies to reduce damage to genetic material and its environmental impact.

## Supplementary Material



## Notes

EDS spectra and semiquantitative elemental composition of ZnO-BK and ZnO-NP samples (Figure S1); statistical analysis of particle size measurements obtained from SEM images for ZnO-BK and ZnO-NP (Table S1); and particle size distribution plots for ZnO-BK and ZnO-NP, including mean particle size values (Figure S2) (PDF)

## Abbreviations

NPs: nanoparticles
ZnO: Zinc Oxide
ZnO-NP: Zinc Oxide nanoparticles
ZnO-BK: Zinc Oxide bulk form
OECD: Organisation for Economic Co-operation and Development
FET: Fish Embryo Acute Toxicity Test
FDA: U.S. Food and Drug Administration
GRAS: Generally Recognized as Safe
ROS: reactive oxygen species
DMSO: dimethyl sulfoxide
DMEM: Dulbecco’s Modified Eagle Medium
FBS: fetal bovine serum
PBS: phosphate-buffered saline
HPF: hours post-fertilization
CONCEA: Brazilian National Council for the Control of Animal Experimentation

## References

[1] International Organization for Standardization (ISO) . Nanotechnologies — Plain language explanation of selected terms from the ISO/IEC 80004 series; ISO/TR 18401:2017. https://www.iso.org/obp/ui/#iso:std:iso:tr:18401:ed-1:v1:en.

[2] Yusuf A., Almotairy A. R. Z., Henidi H., Alshehri O. Y., Aldughaim M. S. (2023). Nanoparticles
as Drug Delivery Systems: A Review of the Implication of Nanoparticles’
Physicochemical Properties on Responses in Biological Systems. Polymers.

[3] Joudeh N., Linke D. (2022). Nanoparticle Classification, Physicochemical Properties, Characterization,
and Applications: A Comprehensive Review for Biologists. J. Nanobiotechnol..

[4] Selmani A., Kovačević D., Bohinc K. (2022). Nanoparticles: From
Synthesis to Applications and Beyond. Adv. Colloid
Interface Sci..

[5] Khan Y., Sadia H., Ali Shah S. Z., Khan M. N., Shah A. A., Ullah N., Ullah M. F., Bibi H., Bafakeeh O. T., Khedher N. Ben., Eldin S. M., Fadhl B. M., Khan M. I. (2022). Classification,
Synthetic, and Characterization Approaches to Nanoparticles, and Their
Applications in Various Fields of Nanotechnology: A Review. Catalysts.

[6] Ijaz I., Gilani E., Nazir A., Bukhari A. (2020). Detail Review on Chemical,
Physical and Green Synthesis, Classification, Characterizations and
Applications of Nanoparticles. Green Chem. Lett.
Rev..

[7] Negrescu A. M., Killian M. S., Raghu S. N. V., Schmuki P., Mazare A., Cimpean A. (2022). Metal Oxide Nanoparticles: Review of Synthesis, Characterization
and Biological Effects. J. Funct. Biomater..

[8] Mandal A. K., Katuwal S., Tettey F., Gupta A., Bhattarai S., Jaisi S., Bhandari D. P., Shah A. K., Bhattarai N., Parajuli N. (2022). Current Research on Zinc Oxide Nanoparticles: Synthesis,
Characterization, and Biomedical Applications. Nanomaterials.

[9] Jiang J., Pi J., Cai J. (2018). The Advancing of Zinc Oxide Nanoparticles for Biomedical
Applications. Bioinorg. Chem. Appl..

[10] Zhou X.-Q., Hayat Z., Zhang D.-D., Li M.-Y., Hu S., Wu Q., Cao Y.-F., Yuan Y. (2023). Zinc Oxide Nanoparticles: Synthesis,
Characterization, Modification, and Applications in Food and Agriculture. Processes.

[11] Lebaka V. R., Ravi P., Reddy M. C., Thummala C., Mandal T. K. (2025). Zinc Oxide
Nanoparticles in Modern Science and Technology: Multifunctional Roles
in Healthcare, Environmental Remediation, and Industry. Nanomaterials.

[12] Xie J., Li H., Zhang T., Song B., Wang X., Gu Z. (2023). Recent Advances
in ZnO Nanomaterial-Mediated Biological Applications and Action Mechanisms. Nanomaterials.

[13] Hazeem L. (2022). Single and
Combined Toxicity Effects of Zinc Oxide Nanoparticles: Uptake and
Accumulation in Marine Microalgae, Toxicity Mechanisms, and Their
Fate in the Marine Environment. Water.

[14] Singh S. (2019). Zinc Oxide
Nanoparticles Impacts: Cytotoxicity, Genotoxicity, Developmental Toxicity,
and Neurotoxicity. Toxicol. Mech. Methods.

[15] Jiménez A., Estudillo E., Guzmán-Ruiz M.
A., Herrera-Mundo N., Victoria-Acosta G., Cortés-Malagón E. M., López-Ornelas A. (2025). Nanotechnology to Overcome Blood–Brain Barrier
Permeability and Damage in Neurodegenerative Diseases. Pharmaceutics.

[16] Song B., Zhang Y., Liu J., Feng X., Zhou T., Shao L. (2016). Is Neurotoxicity of Metallic Nanoparticles the Cascades of Oxidative
Stress?. Nanoscale Res. Lett..

[17] Chen C., Pei X., Yu Y., Gao C., Wang J., Zhu R., Liu S., Tang S., Li D. (2026). Zinc Oxide Nanoparticle-Induced Neurotoxicity:
Underlying Molecular Mechanisms and Future Perspectives. Toxics.

[18] Abdelmeged E. A., Brunetti G., Shetaya W. H., Marzouk E. R. (2023). Cosmetic Nanomaterials
in the Environment: Nano-Zinc Oxide and Zinc-Influence on Soil Microorganisms. Appl. Nanosci..

[19] Salami M., Khosravi M., Zarei M. H. (2022). Comparative Toxic Effect of Bulk
Zinc Oxide (ZnO) and ZnO Nanoparticles on Human Red Blood Cells. Main Group Met. Chem..

[20] OECD . Test No. 471: Bacterial Reverse Mutation Test; OECD Guidelines for the Testing of Chemicals, Section 4; OECD, 2020.

[21] OECD . Test No. 487: In Vitro Mammalian Cell Micronucleus Test; OECD Guidelines for the Testing of Chemicals, Section 4; OECD, 2023.

[22] OECD . Test No. 236: Fish Embryo Acute Toxicity (FET) Test; OECD Guidelines for the Testing of Chemicals, Section 2; OECD Publishing: Paris, 2025 10.1787/9789264203709-en.

[23] Kermanizadeh A., Powell L. G., Stone V. (2020). A Review of Hepatic Nanotoxicology
– Summation of Recent Findings and Considerations for the next
Generation of Study Designs. J. Toxicol. Environ.
Heal. Part B.

[24] Lourenço J., Marques S., Carvalho F. P., Oliveira J., Malta M., Santos M., Gonçalves F., Pereira R., Mendo S. (2017). Uranium Mining
Wastes: The Use of the Fish Embryo Acute Toxicity Test (FET) Test
to Evaluate Toxicity and Risk of Environmental Discharge. Sci. Total Environ..

[25] Lin L.-Y., Kantha P., Horng J.-L. (2023). Toxic Effects of Polystyrene Nanoparticles
on the Development, Escape Locomotion, and Lateral-Line Sensory Function
of Zebrafish Embryos. Comp. Biochem. Physiol.
Part C Toxicol. Pharmacol..

[26] de
Souza I. R., de Oliveira J. B. V., Sivek T. W., de Albuquerque
Vita N., Canavez A. D. P. M., Schuck D. C., Cestari M. M., Lorencini M., Leme D. M. (2024). Prediction of Acute Fish Toxicity
(AFT) and Fish Embryo Toxicity (FET) Tests by Cytotoxicity Assays
Using Liver and Embryo Zebrafish Cell Lines (ZFL and ZEM2S). Chemosphere.

[27] Hariharan S., Chauhan S., Marcharla E., Alphonse C. R. W., Rajaretinam R. K., Ganesan S. (2024). Developmental Toxicity and Neurobehavioral Effects
of Sodium Selenite and Selenium Nanoparticles on Zebrafish Embryos. Aquat. Toxicol..

[28] Hsiao B.-Y., Horng J.-L., Yu C.-H., Lin W.-T., Wang Y.-H., Lin L.-Y. (2024). Assessing Cardiovascular Toxicity in Zebrafish Embryos
Exposed to Copper Nanoparticles. Comp. Biochem.
Physiol. Part C Toxicol. Pharmacol..

[29] Schindelin J., Arganda-Carreras I., Frise E., Kaynig V., Longair M., Pietzsch T., Preibisch S., Rueden C., Saalfeld S., Schmid B., Tinevez J.-Y., White D. J., Hartenstein V., Eliceiri K., Tomancak P., Cardona A. (2012). Fiji: An Open-Source
Platform for Biological-Image Analysis. Nat.
Methods.

[30] Maron D. M., Ames B. N. (1983). Revised Methods for the Salmonella Mutagenicity Test. Mutat. Res. Mutagen. Relat. Subj..

[31] Carrão
Dantas E. K., Ferreira C. L. S., da Cunha Goldstein A., da Silva Fernandes A., Anastacio Ferraz E. R., Felzenszwalb I., Araújo-Lima C. F. (2024). Marketable 1,3-Dimethylamylamine
and Caffeine-Based Thermogenic Supplements: Regulatory Genotoxicity
Assessment through in Vitro and in Silico Approaches. J. Toxicol. Environ. Health Part A.

[32] de
Oliveira R. T., da Silva Oliveira J.
P., da Silva A. L. M., Carrão Dantas E. K., Koblitz M. G. B., Bello M. L., Felzenszwalb I., Araújo-Lima C.
F., Macedo A. F. (2024). Vanilla
from Brazilian Atlantic Forest: In Vitro and in Silico Toxicity Assessment
and High-Resolution Metabolomic Analysis of Vanilla Spp. Ethanolic
Extracts. Food Chem..

[33] Santhosh P. B., Hristova-Panusheva K., Petrov T., Stoychev L., Krasteva N., Genova J. (2024). Femtosecond Laser-Induced Photothermal Effects of Ultrasmall
Plasmonic Gold Nanoparticles on the Viability of Human Hepatocellular
Carcinoma HepG2 Cells. Cells.

[34] Carrão
Dantas E. K., Thomé J. P. F.
T., Pontes Filho M. de O., Coelho M. G. P., Felzenszwalb I., Araujo-Lima C. F. (2026). Multi-Herbal
Ergogenic Supplement Induces in Vitro Mutagenicity and Cytogenetic
Damages in HepG2 and HEK293 Cells. Mutagenesis.

[35] Maciel-Magalhães M., Medeiros R. J., Guedes N. C. do C., Brito T. M. de., Souza G. F. de., Canabarro B. R., Ferraris F. K., Amendoeira F. C., Rocha H. V. A., Patricio B. F. de C., Delgado I. F. (2025). Amphotericin B Encapsulation
in Polymeric Nanoparticles: Toxicity Insights via Cells and Zebrafish
Embryo Testing. Pharmaceutics.

[36] Westerfield, M. The Zebrafish Book. A Guide for the Laboratory Use of Zebrafish (Danio Rerio). 4th ed.; Univ. of Oregon Press: OR, 2000.

[37] Wang X., Sun T., Zhu H., Han T., Wang J., Dai H. (2020). Roles of PH,
Cation Valence, and Ionic Strength in the Stability and Aggregation
Behavior of Zinc Oxide Nanoparticles. J. Environ.
Manage..

[38] de
O Primo J., Bittencourt C., Acosta S., Sierra-Castillo A., Colomer J.-F., Jaerger S., Teixeira V. C., Anaissi F. J. (2020). Synthesis
of Zinc Oxide Nanoparticles by Ecofriendly Routes: Adsorbent for Copper
Removal From Wastewater. Front. Chem..

[39] Meißner T., Oelschlägel K., Potthoff A. (2014). Implications of the Stability Behavior
of Zinc Oxide Nanoparticles for Toxicological Studies. Int. Nano Lett..

[40] Herrmann R., García-García F. J., Reller A. (2014). Rapid Degradation of
Zinc Oxide Nanoparticles by Phosphate Ions. Beilstein J. Nanotechnol..

[41] Wells M. A., Abid A., Kennedy I. M., Barakat A. I. (2012). Serum Proteins Prevent
Aggregation of Fe 2 O 3 and ZnO Nanoparticles. Nanotoxicology.

[42] Yu J., Kim H.-J., Go M.-R., Bae S.-H., Choi S.-J. (2017). ZnO Interactions
with Biomatrices: Effect of Particle Size on ZnO-Protein Corona. Nanomaterials.

[43] Vaisman A., McDonald J. P., Woodgate R. (2012). Translesion DNA Synthesis. EcoSal Plus.

[44] Mortelmans K., Zeiger E. (2000). The Ames Salmonella/Microsome Mutagenicity Assay. Mutat. Res. Mol. Mech. Mutagen..

[45] Yu, G. ; Guo, Z. ; Li, H. Environmental Toxicology and Toxicogenomics; Pan, X. ; Zhang, B. , Eds.; Springer US: New York, NY, 2021; Vol. 2326.

[46] Pan X., Redding J. E., Wiley P. A., Wen L., McConnell J. S., Zhang B. (2010). Mutagenicity Evaluation of Metal Oxide Nanoparticles by the Bacterial
Reverse Mutation Assay. Chemosphere.

[47] Li J., Chang X., Chen X., Gu Z., Zhao F., Chai Z., Zhao Y. (2014). Toxicity of Inorganic Nanomaterials
in Biomedical Imaging. Biotechnol. Adv..

[48] Shen C., James S. A., de Jonge M. D., Turney T. W., Wright P. F. A., Feltis B. N. (2013). Relating Cytotoxicity, Zinc Ions, and Reactive Oxygen
in ZnO Nanoparticle–Exposed Human Immune Cells. Toxicol. Sci..

[49] do
Carmo Neto J. R., Franco P. I. R., Braga Y. L. L., de
Oliveira J. F., Perini H. F., Albuquerque L. F. D., Martins D. B., Helmo F. R., Andrade A. A., Miguel M. P., Celes M. R. N., Rocha T. L., Almeida Silva A. C., Machado J. R., da Silva M. V. (2024). Toxicity Assessment of New Ag-ZnO/AgO
Nanocomposites: An In Vitro and In Vivo Approach. J. Funct. Biomater..

[50] Belal R., Gad A. (2023). Zinc Oxide Nanoparticles Induce Oxidative Stress, Genotoxicity, and
Apoptosis in the Hemocytes of Bombyx Mori Larvae. Sci. Rep..

[51] Syama S., Reshma S. C., Sreekanth P. J., Varma H. K., Mohanan P. V. (2013). Effect
of Zinc Oxide Nanoparticles on Cellular Oxidative Stress and Antioxidant
Defense Mechanisms in Mouse Liver. Toxicol.
Environ. Chem..

[52] Ickrath P., Wagner M., Scherzad A., Gehrke T., Burghartz M., Hagen R., Radeloff K., Kleinsasser N., Hackenberg S. (2017). Time-Dependent Toxic and Genotoxic Effects of Zinc
Oxide Nanoparticles after Long-Term and Repetitive Exposure to Human
Mesenchymal Stem Cells. Int. J. Environ. Res.
Public Health.

[53] Olejnik M., Kersting M., Rosenkranz N., Loza K., Breisch M., Rostek A., Prymak O., Schürmeyer L., Westphal G., Köller M., Bünger J., Epple M., Sengstock C. (2021). Cell-Biological Effects of Zinc Oxide
Spheres and Rods from the Nano- to the Microscale at Sub-Toxic Levels. Cell Biol. Toxicol..

[54] Cardoso D., Narcy A., Durosoy S., Bordes C., Chevalier Y. (2021). Dissolution
Kinetics of Zinc Oxide and Its Relationship with Physicochemical Characteristics. Powder Technol..

[55] Mu S., Yang H., Wang S., Tong A., Ding R., Wang J., Wang D., Li J. (2025). Zinc-Based Nanomaterials
in Cancer Therapy: Mechanisms, Applications, and Future Directions. Theranostics.

[56] Araujo-Lima C. F., Nunes R. J. M., Carpes R. M., Aiub C. A. F., Felzenszwalb I. (2017). Pharmacokinetic
and Toxicological Evaluation of a Zinc Gluconate-Based Chemical Sterilant
Using In Vitro and In Silico Approaches. Biomed.
Res. Int..

[57] Xia T., Zhao Y., Sager T., George S., Pokhrel S., Li N., Schoenfeld D., Meng H., Lin S., Wang X., Wang M., Ji Z., Zink J. I., Mädler L., Castranova V., Lin S., Nel A. E. (2011). Decreased Dissolution
of ZnO by Iron Doping Yields Nanoparticles with Reduced Toxicity in
the Rodent Lung and Zebrafish Embryos. ACS Nano.

[58] Böhme S., Baccaro M., Schmidt M., Potthoff A., Stärk H.-J., Reemtsma T., Kühnel D. (2017). Metal Uptake and Distribution in
the Zebrafish (Danio Rerio) Embryo: Differences between Nanoparticles
and Metal Ions. Environ. Sci. Nano.

[59] Cheng J., Flahaut E., Cheng S. H. (2007). Effect of Carbon Nanotubes on Developing
Zebrafish (Danio Rerio) Embryos. Environ. Toxicol.
Chem..

[60] Kim K.-T., Tanguay R. L. (2014). The Role of Chorion on Toxicity of Silver Nanoparticles
in the Embryonic Zebrafish Assay. Environ. Health
Toxicol..

[61] Rawson D. M., Zhang T., Kalicharan D., Jongebloed W. L. (2000). Field Emission
Scanning Electron Microscopy and Transmission Electron Microscopy
Studies of the Chorion, Plasma Membrane and Syncytial Layers of the
Gastrula-Stage Embryo of the Zebrafish Brachydanio Rerio: A Consideration
of the Structural and Functional. Aquacult.
Res..

[62] Zhu X., Zhu L., Duan Z., Qi R., Li Y., Lang Y. (2008). Comparative
Toxicity of Several Metal Oxide Nanoparticle Aqueous Suspensions to
Zebrafish (Danio Rerio) Early Developmental Stage. J. Environ. Sci. Health Part A.

[63] Zhao X., Wang S., Wu Y., You H., Lv L. (2013). Acute ZnO
Nanoparticles Exposure Induces Developmental Toxicity, Oxidative Stress
and DNA Damage in Embryo-Larval Zebrafish. Aquat.
Toxicol..

[64] Yu L.-p., Fang T., Xiong D., Zhu W., Sima X. (2011). Comparative
Toxicity of Nano-ZnO and Bulk ZnO Suspensions to Zebrafish and the
Effects of Sedimentation, ˙OH Production and Particle Dissolution
in Distilled Water. J. Environ. Monit..

[65] Choi J. S., Song J., Yoon S., Kim W.-K. (2018). Predicting ZnO Nanoparticle
and ZnSO4 Toxicity from Zebrafish Embryo Gene Expression. Mol. Cell. Toxicol..

[66] Lee Y.-L., Shih Y.-S., Chen Z.-Y., Cheng F.-Y., Lu J.-Y., Wu Y.-H., Wang Y.-J. (2022). Toxic Effects and Mechanisms of Silver
and Zinc Oxide Nanoparticles on Zebrafish Embryos in Aquatic Ecosystems. Nanomaterials.

[67] Takesono A., Dimitriadou S., Clark N. J., Handy R. D., Mourabit S., Winter M. J., Kudoh T., Tyler C. R. (2023). Zinc Oxide Nanoparticles
Disrupt Development and Function of the Olfactory Sensory System Impairing
Olfaction-Mediated Behaviour in Zebrafish. Environ.
Int..

[68] Wei X., Xu G., Wang Z. (2025). Induction of Zebrafish Embryotoxicity by Zinc Oxide
Nanoparticles: A Meta-Analysis. J. Nanopart.
Res..

[69] Hoyberghs J., Bars C., Ayuso M., Van Ginneken C., Foubert K., Van Cruchten S. (2021). DMSO Concentrations up to 1% Are
Safe to Be Used in the Zebrafish Embryo Developmental Toxicity Assay. Front. Toxicol..

[70] Christou M., Kavaliauskis A., Ropstad E., Fraser T. W. K. (2020). DMSO Effects
Larval Zebrafish (Danio Rerio) Behavior, with Additive and Interaction
Effects When Combined with Positive Controls. Sci. Total Environ..

[71] Kais B., Schneider K. E., Keiter S., Henn K., Ackermann C., Braunbeck T. (2013). DMSO Modifies the Permeability of the Zebrafish (Danio
Rerio) Chorion-Implications for the Fish Embryo Test (FET). Aquat. Toxicol..

[72] Gomes G., de Sousa Andrade A. C., de Carvalho Vieira P., Spineli M. N., Costa M. L., Mermelstein C. (2025). DMSO Induces Major Morphological and Physiological
Alterations in Zebrafish Embryos. PLoS One.

[73] Zhu X., Wang J., Zhang X., Chang Y., Chen Y. (2009). The Impact
of ZnO Nanoparticle Aggregates on the Embryonic Development of Zebrafish
(Danio Rerio). Nanotechnology.

[74] Bai W., Zhang Z., Tian W., He X., Ma Y., Zhao Y., Chai Z. (2010). Toxicity of Zinc Oxide Nanoparticles
to Zebrafish Embryo: A Physicochemical Study of Toxicity Mechanism. J. Nanopart. Res..

[75] Hua J., Vijver M. G., Richardson M. K., Ahmad F., Peijnenburg W. J. G. M. (2014). Particle-Specific
Toxic Effects of Differently Shaped Zinc Oxide Nanoparticles to Zebrafish
Embryos (Danio Rerio. Environ. Toxicol. Chem..

[76] Brun N. R., Lenz M., Wehrli B., Fent K. (2014). Comparative Effects
of Zinc Oxide Nanoparticles and Dissolved Zinc on Zebrafish Embryos
and Eleuthero-Embryos: Importance of Zinc Ions. Sci. Total Environ..

[77] Kteeba S. M., El-Adawi H. I., El-Rayis O. A., El-Ghobashy A. E., Schuld J. L., Svoboda K. R., Guo L. (2017). Zinc Oxide Nanoparticle
Toxicity in Embryonic Zebrafish: Mitigation with Different Natural
Organic Matter. Environ. Pollut..

