# Supplementary material for: A Comparative Analysis of Zinc Oxide as Nanoparticles and Its Bulk Form through In Vitro Toxicogenetics and In Vivo Zebrafish Embryotoxicity Models
Source: ACS Omega. 2026 Sep 22;11(39):60104–18. doi: 10.1021/acsomega.6c07375 (PMC13647433; doi:10.1021/acsomega.6c07375)
Supplement: Supplementary file 1 [file ao6c07375_si_001.pdf]

Supporting Information

A comparative analysis of zinc oxide as nanoparticles and its bulk form through *in vitro* toxicogenetics and *in vivo* zebrafish embryotoxicity models

Carlos Fernando Araujo-Lima [1,2,3,4,a], Magno Maciel-Magalhães [5,a,] *, Marcela Correia da Rocha Honorato [1,2,a], Hana de Jesús Galiza Gomes [1,2], Pedro Paulo Saldanha Coimbra [1,2], Nayara Cecília do Couto Guedes [5], Thiago dos Santos Cabral-Rocha [3,5], Lays Souza [2], Bárbara Verena Dias Galvão [2], Beatriz Rodrigues Canabarro [6], Gabriele Fátima de Souza [5], Thais Morais de Brito [5], Otniel Freitas-Silva [4,7], Renata Jurema Medeiros [5], Beatriz Ferreira de Carvalho Patrício [1,3], Israel Felzenszwalb [2]

AUTHORS' AFFILIATIONS

[1] Universidade Federal do Estado do Rio de Janeiro (UNIRIO), Laboratório Integrado de Mutagênese Ambiental (LIMA), Rio de Janeiro 20211-010, RJ, Brazil.

[2] Universidade do Estado do Rio de Janeiro (UERJ), Laboratório de Mutagênese Ambiental (LABMUT), Rio de Janeiro 20551-030, RJ, Brazil.

[3] Universidade Federal do Estado do Rio de Janeiro (UNIRIO), Laboratório de Inovação Farmacêutica e Tecnológica (LIFT), Rio de Janeiro 20211-010, RJ, Brazil.

[4] Universidade Federal do Estado do Rio de Janeiro (UNIRIO), Programa de Pós-Graduação em Alimentos e Nutrição (PPGAN), Rio de Janeiro 20211-010, RJ, Brazil

[5] Fundação Oswaldo Cruz (FIOCRUZ), Instituto Nacional de Controle de Qualidade em Saúde (INCQS), Plataforma de Ensaios Toxicológicos em Peixes (FIOFISHTOX), Rio de Janeiro 21040-900, Brazil.

[6] Centro Brasileiro de Pesquisas Físicas (CBPF), Rio de Janeiro 22290-180, Brazil.

[7] Empresa Brasileira de Pesquisa Agropecuária (EMBRAPA), Rio de Janeiro 23020-470, RJ, Brazil.

* Corresponding author

[a] These authors contributed equally to this study.

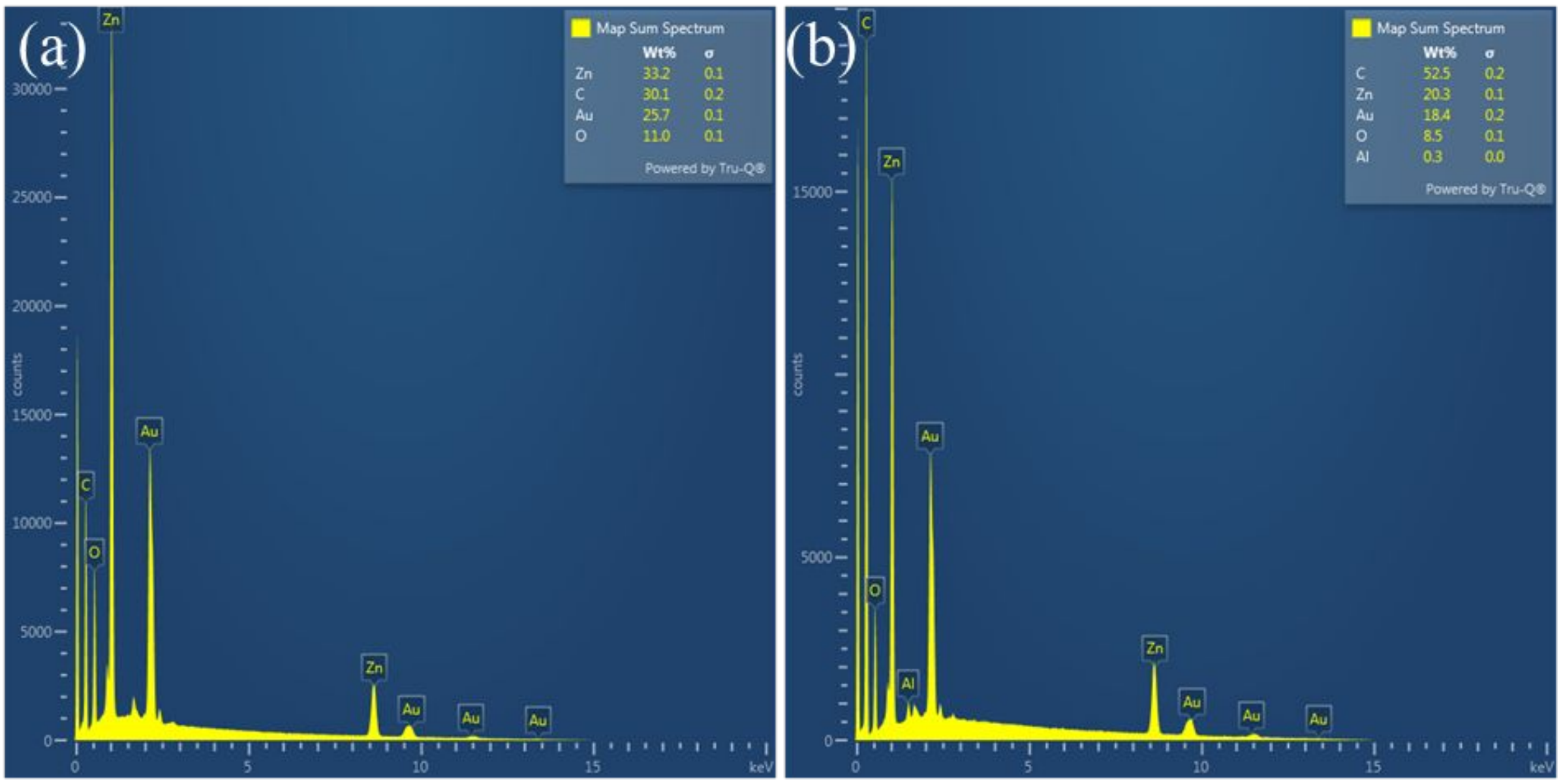

Figure S1. EDS spectrum of ZnO-BK (a), and ZnO-NP (b), with semi-quantitative chemical composition, and the SEM image of the analyzed region in the inset.

Table S1. Statistics of particle size measurement of both samples using SEM images

Sample	n	Mean ± SD (µm)	Min (µm)	Max (µm)	Median (µm)
ZnO-NP	227	0.078 ± 0.048	0.026	0.351	0.069
ZnO-BK	227	0.275 ± 0.125	0.078	0.721	0.252

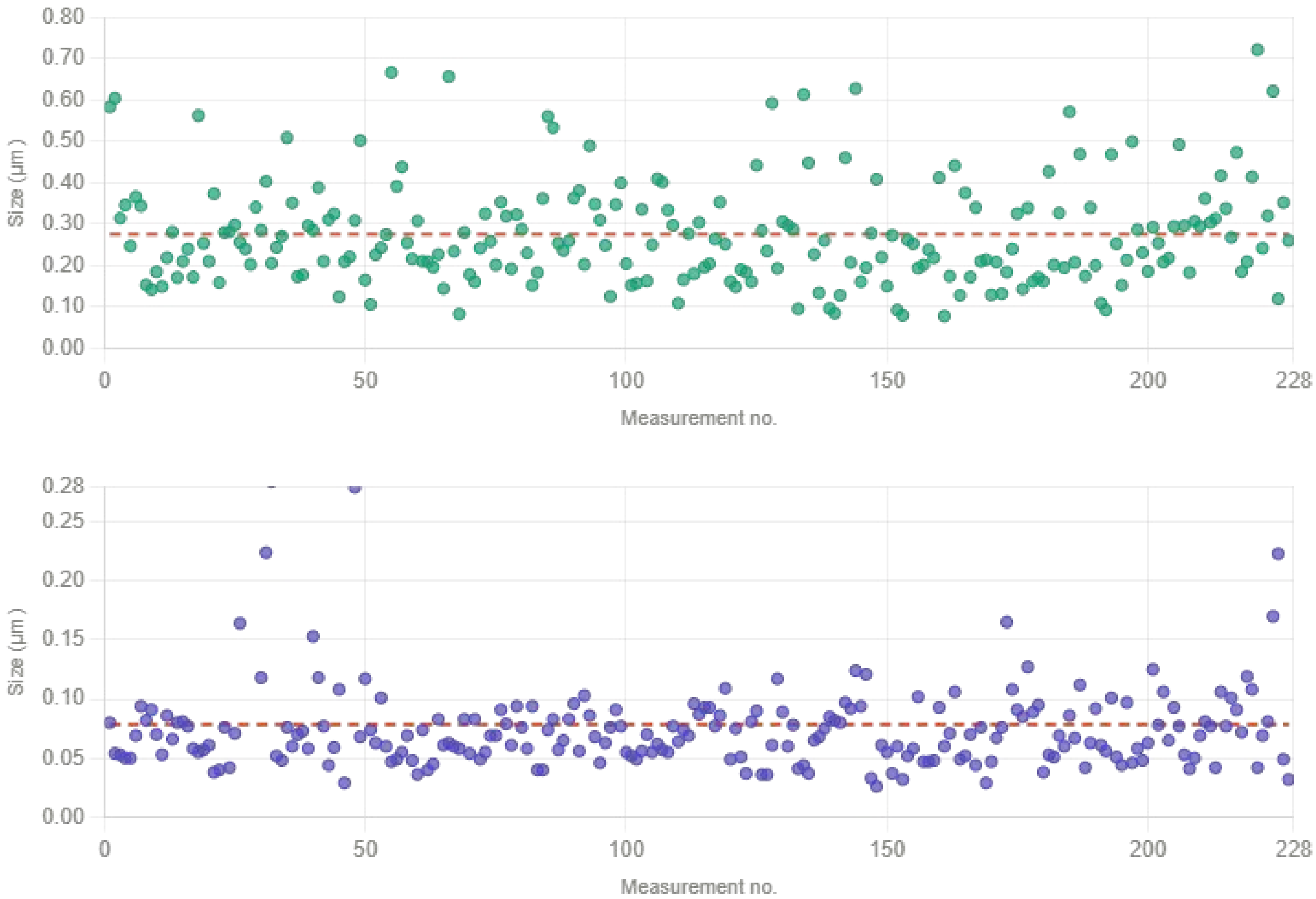

Figure S2. Dispersion of particle size measurement of ZnO-BK (green), and ZnO-NP (purple). The mean value is marked in a red dashed line in both graphs.
